# Acceptability of Nonpharmaceutical Interventions to Prevent the Risk of COVID-19 Infection in the United States

**DOI:** 10.1177/23814683261455894

**Published:** 2026-06-24

**Authors:** Rachel E. Murray-Watson, Marcy Ekanayake-Weber, Ted Cohen, Janel Hanmer, Reza Yaesoubi

**Affiliations:** Department of Infectious Disease Epidemiology, School of Public Health, Imperial College London, London, UK; Department of Anthropology, University at Albany, Albany, NY, USA; Department of Epidemiology of Microbial Diseases, Yale School of Public Health, New Haven, CT, USA; Division of General Internal Medicine, University of Pittsburgh School of Medicine, Pittsburgh, PA, USA; Philip R. Lee Institute for Health Policy Studies, University of California San Francisco, San Francisco, CA, USA

**Keywords:** nonpharmaceutical interventions, pandemics, discrete-choice experiments, preference, utility

## Abstract

**Background**. Nonpharmaceutical interventions (NPIs) were critical for mitigating COVID-19 transmission, yet their social and economic costs generated substantial public resistance. We aimed to quantify US adults’ acceptance of key NPIs. **Methods**. We conducted a national discrete-choice experiment between May and December 2024 among a quota-based panel of US adults, considering 6 NPIs: mask mandates, reduced public transit capacity, school closures with/without remote learning, closure of crowded indoor venues, and closure of nonessential businesses (e.g., restaurants). Scenarios varied by COVID-19 infection risk and health care restrictions. **Results**. A total of 5,047 adults participated, with age, gender, race, and income distributions reflecting those of the US population. Participants considered mask mandates, reduced transit capacity, and school closures with remote learning acceptable even if they do not reduce cases. School closures without remote learning and closure of nonessential businesses were acceptable only if they reduced cases by at least 75 per 100 persons within 1 mo. Restricted access to primary and optional care was strongly objected to. Male participants, Republicans, Independents, adults ≥65 y, and those without chronic conditions showed the lowest acceptance of NPIs regardless of projected benefit. Female participants, younger adults, those living with children, and those with chronic conditions were more receptive. Still, they required a reduction of >50 cases per 100 persons to accept school closures without remote learning and closure of nonessential businesses. **Limitations**. Temporal distancing may have affected participants’ recollection of their experiences with NPIs. **Conclusions**. US adults displayed heterogeneous but generally high thresholds for accepting school closures without remote learning, closure of nonessential businesses, and health care restrictions. These findings can guide pandemic preparedness plans that balance public acceptance with disease-control goals.

**Highlights:**

- What levels of COVID-19 risk reduction were required for US adults to accept nonpharmaceutical interventions (NPIs) such as school or business closures?
- In a discrete-choice experiment of 5,047 US adults, mask mandates and other moderate NPIs were broadly acceptable even if they do not reduce cases. School closures without remote learning and closure of nonessential businesses were acceptable only if they reduced infections by ≥75 per 100 persons per month.
- Our survey participants did not find pandemic response plans that rely on disruptive NPIs acceptable but found indoor mask mandates and school closures with remote learning acceptable, independent of their impact on infection risk.

Large-scale implementation of various nonpharmaceutical interventions (NPIs), such as school and business closure, was used to curb the spread of SARS-CoV-2 and to mitigate surges in the COVID-19–related hospitalizations and deaths.^1,2^ The extended use of NPIs that rely on strict physical distancing (e.g., school and business closure) may adversely affect other outcomes due to losses of income and health insurance, increases in mental health issues and substance use disorders, interruptions in scheduled health care visits, and educational delays.^3–9^ These adverse outcomes are often concentrated among economically disadvantaged groups, people with disabilities, and the elderly; hence, the prolonged use of such NPIs may also exacerbate the challenges these groups face.^3,4,10–15^

Despite these challenges, NPIs will most likely continue to play a role in pandemic response, particularly before effective vaccines are available. Yet, little is known about the US population’s preferences for different NPIs and their willingness to accept (WTA) the health and financial consequences of NPIs to mitigate pandemic-related adverse health outcomes. Quantifying population preferences assists policy makers in balancing the health benefits and negative impacts of NPIs and allows for the design of control measures that are most likely to be accepted and adhered to in target populations.^16,17^

Our study aimed to elicit the US population’s preference for using different NPIs to mitigate COVID-related health outcomes and to investigate whether preferences differ based on demographic, socioeconomic, and clinical characteristics (e.g., age, gender, political affiliation, living with chronic conditions, and residence in urban, suburban, or rural areas). To this end, we conducted discrete-choice experiments (DCEs) using a sample of adult US participants, recruited between May and December 2024 from a quota-based US internet panel. DCEs are commonly used in health, transportation, and environmental domains to collect evidence regarding the population’s preferences to support policy making.^18–20^ Using DCEs, we also estimate the population’s WTA NPIs, defined as the minimum effectiveness, in reducing infections over the next month, that an NPI must achieve to be acceptable to a population. We estimate these WTA values among people with different sociodemographic, clinical, and economic backgrounds.

## Methods

We designed DCEs to quantify individuals’ preferences for different NPIs (e.g., school and business closure) alongside COVID-related outcomes (e.g., infection rate). DCE is a survey methodology based on random utility theory to gauge respondents’ preferences over different, and often conflicting, outcomes.^21^ It achieves this by presenting respondents with a series of alternative scenarios from which they must choose one. These scenarios are characterized by a range of “attributes” that could take different “levels.” Below, we describe the attributes and levels we used to design our DCE.

### Attributes and Levels

To choose attributes and levels that define pandemic scenarios in our survey, we first reviewed the range of NPIs implemented in the United States between March 2020 and May 2023,^22–24^ along with a literature search of other DCEs focused on NPIs for COVID-19.^22–25^ After multiple rounds of expert elicitation involving public health officials, epidemiologists, and population health researchers, we selected 4 categories of NPIs and 2 categories of health outcomes as attributes of our DCEs (Table 1). When selecting attributes, we focused on those that would be easily understood by a broad audience, had been implemented across different pandemic settings, and could be used in future responses to infectious disease threats.

**Table 1:** Attributes and Levels to Define Pandemic Scenarios in our Discrete-Choice Experiments

Attribute	Levels
Mask mandates	1. No mask mandate in public settings 2. Mask mandates in schools from kindergarten to 12th grade 3. Mask mandates in public indoor spaces such as shops, offices, and theatres, but not in schools 4. Mask mandates in all public indoor spaces and schools from kindergarten to 12th grade
School closures	1. No schools close 2. Only high schools are closed, but remote learning is available 3. Only high schools are closed, but remote learning is not available 4. All schools are closed, but remote learning is available 5. All schools are closed, but remote learning is not available
Business closures	1. No businesses close 2. Crowded indoor event venues, such as clubs and theaters, close 3. All nonessential businesses, such as restaurants and gyms, close
Reduction in public transit capacity	1. No reduction in public transit capacity 2. 50% reduction in public transit capacity
Number of people infected over the next month	1. 1 in 100 people 2. 5 in 100 people 3. 10 in 100 people 4. 15 in 100 people 5. 20 in 100 people
Access to health care	1. No restrictions 2. Access to primary care is limited (routine care or preventative care, such as checkups and treatment for nonurgent illnesses would be canceled, delayed, or provided by telemedicine) 3. Access to primary care and optional procedures (such as colonoscopy and cataract surgery) is limited, and health care is available only in emergencies

We considered 4 categories of NPIs: 1) mask mandates, 2) school closures, 3) business closures, and 4) reduction in public transit capacity. Each of these interventions can be implemented at different levels, which are listed in Table 1. We included 2 health-related attributes: 1) the number of people infected over the next month and 2) restrictions on access to health care over the next month. By including both NPIs and health-related attributes, we could characterize more realistic, tangible pandemic scenarios that capture the different ways the COVID-19 pandemic affected the day-to-day lives of the US population. This also allows us to evaluate whether preferences for NPIs change with their effectiveness at reducing infections and ensuring access to health care.

For reasons of pragmatism and in line with ISPOR’s good research practice guidelines,^26^ the levels of each attribute we consider are nonexhaustive but do capture the range of intensities of NPIs employed during the federally declared public health emergency between March 2020 and May 2023.^22–25^ Details on how these attributes were presented to participants are described in Supplementary Table S4.

### Accounting for the Impact of Vaccine Availability

Since the availability of an effective vaccine could alter the population’s preferences for different NPIs, we fielded 2 versions of our DCE. In one version, participants were asked to consider a scenario in which no vaccine is available, and consequently, 5 in 100 infected people would require hospitalization. In the second version, participants were asked to consider the scenario if a vaccine were available and 1 in 100 infected people would require hospitalization. There was no overlap between the respondents, and participating in one survey prevented invitation to the other.

### Survey Design

We employed a pairwise DCE design, in which participants were asked to repeatedly choose their preferred option from 2 hypothetical scenarios (see Supplementary Figure S1 for an example). We designed these choice tasks using Qualtrics Conjoint Analysis Software. Given the many possible choice tasks, we partitioned the tasks into 250 blocks, randomly distributed across respondents^27^ (§S1.1). Blocking allows us to reduce the number of questions given to each participant while maintaining a balance of attribute levels across all blocks. Qualtrics Conjoint Analysis Software designed each block with a randomized balance, ensuring that each attribute level within a particular block is shown approximately the same number of times. In addition to a written summary of each attribute level, we included a pictorial representation (“icons”) of the attribute level. These icons are shown in Supplementary Table S4. We manually prohibited certain combinations of nonsensical levels, namely, any combination that required masking in schools and closure of all schools.

Before fielding the surveys, we pilot tested the survey introductory material, choice experiments, and descriptions of NPIs among more than 15 people outside the investigative team with varying sociodemographic, clinical vulnerability, and economic vulnerability backgrounds. Empirical studies suggest that testing surveys/user interfaces on 10 participants is expected to detect >80% of usability problems.^28–30^ The pilot test used think-aloud methods and cognitive interviewing.^31^ The results were used to revise instructions, icons, and task wording. We conducted another pilot test until no major revisions were needed.

In line with the recommendations made by the ISPOR good research practices guideline,^32^ each survey respondent received 10 choice tasks. Because in real-world situations, an individual is unlikely to opt out of a county where decisions about mitigation strategies must be made, we required each participant to choose 1 of the 2 presented scenarios (i.e., “none of the above” option was not provided; Supplementary Figure S1).

Each participant was also required to answer supplementary questions on socioeconomic, demographic, and health factors (Supplementary Table S2). We chose these questions based on literature describing known inequalities in the United States and assumptions about what characteristics may influence a participant’s preferences.^3,10–12,15,24,25^

### Sample Size

Our minimum sample size calculation was calculated using Johnson and Orme’s Rule of Thumb,^33,34^ given by $N>\frac{500c}{\mathrm{ta}}$ , where N is the minimum sample size for each version of the survey, c is the largest number of levels for any attribute when considering only main effects,^5^ a is the number of alternatives from which to choose,^2^ and t is the number of choice tasks.^10^ This gives us a minimum sample size of $\frac{500c\times 5}{10\times 2}=125$ . However, as this was a small sample size, and we were interested in the experiences of diverse populations, we multiplied this by a factor of 20 to arrive at N=2,500 for each version of our survey.

### Survey Population

We used the online survey platform Qualtrics to recruit an online panel and administer our survey (for details on Qualtrics’ recruitment process, see Qualtrics ESOMAR^35^). The sample consisted of adults aged ≥18 y who were residents of the United States between March 2020 and July 2022 and understood written and oral English. The panel was recruited to meet specific quotas on the distribution of age, gender, race, and household income to ensure representativeness based on the general US adult population (Supplementary Table S1). The specified quotas were determined based on the US Census data.^36^

Questions on the participants’ socioeconomic, demographic, and health factors were asked upon completion of the DCE. These were included after completing the DCE task to mitigate any potential effects of identity priming.^37,38^ Qualtrics offered each participant a reward in accordance with its internal guidelines upon completing the survey.^35^ All survey participants remained anonymous, and no personally identifiable information was collected.

We conducted an initial analysis with a sample size of 200 per survey to assess user experience and data quality. To this end, we allowed participants to provide comments at the end of the survey about their experience, checked if the distribution of participants across quota groups is close to the specified quota (Supplementary Table S1), and conducted the statistical analysis described below using the pilot data to detect any anomalies. These pilot studies did not identify any specific issues. Hence, we also included the data collected as part of pilot studies in the final dataset.

### Statistical Analysis

We estimated the utilities of each level of NPIs and COVID-related health outcomes using a mixed logit model (also called the random parameters logit model).^21,39^ These models assume that the likelihood of selecting a profile from a set of alternatives is a function of the attribute levels defining the alternatives, along with a random error term that accounts for individual-specific variations in utilities. Using this model, we can estimate both the mean preference weights and the distribution of preference weights across the sample population.^25^

We assumed that the utility of the individual n under scenario j described in the choice task t can be modeled as

$$U_{\mathrm{njt}}=\alpha_{n}S_{\mathrm{nt}}+\beta_{n}^{\prime }x_{\mathrm{njt}}+\epsilon_{\mathrm{njt}}$$

where $x_{\mathrm{njt}}\;$ are observed variables related to the scenario j presented to the participant n in a choice task t and $\beta_{n}$ is the vector of coefficients for these variables for participant n . The error term $\epsilon_{\mathrm{njt}}$ follows a Gumbel distribution (or extreme value type 1 distribution), in line with the assumptions of a mixed logit model.^21^ The error term accounts for unobserved factors that may influence an individual’s utility. We included a constant, $S_{\mathrm{nt}}$ , and its associated coefficient $\alpha_{n}$ , for selecting the scenario presented in the right panel, irrespective of its attribute levels.^25^ This helps account for any effects of the survey layout on participants’ responses. We estimated coefficients for attribute levels relative to the baseline, defined as no NPIs in effect and normal access to health care. The variable representing the infection rate is continuous. The remaining variables representing NPI levels and access to health care were dummy coded (see §S1.2).

To quantify the tradeoffs between the number of cases and the use of NPIs, we also calculated the WTA thresholds. For NPI k that is implemented at the level l , the WTA is defined as $\beta_{\mathrm{kl}}/\beta_{I}$ , where $\beta_{\mathrm{kl}}$ is the estimated main effect for NPI k if implemented at the level l , and $\beta_{I}$ is the estimated main effect for infection rate during the next month.^40^ For a given level of an NPI, the WTA value estimates the minimum reduction in the number of cases during the next month for an average individual to accept the introduction of the NPI at that specific level (see §S1.3 for additional information).

To estimate heterogeneity in the perceived utility across different population groups, we conducted subgroup analyses by estimating the mixed logit model described above for select demographic and socioeconomic groups (Supplementary Table S2). To this end, we fit a mixed logit model using data from a specific subgroup (e.g., individuals younger than 65 y) and report the subgroup’s WTA thresholds. We repeat this process for subgroups defined by age, gender, living with chronic conditions, political affiliation, race, having school-aged children, having vulnerable contact, and residing in urban, suburban, or rural areas. The models were estimated using Biogeme v3.2.13.^41^

## Results

In total, 2,519 and 2,527 participants completed the surveys for “vaccine” and “no vaccine” scenarios between May and December 2024. The demographic and socioeconomic characteristics of participants were similar between both surveys, with the age, gender, race, and income distribution of the study sample reflecting that of the US population (Table S1–Table S3). Under “vaccine” and “no vaccine” survey scenarios, 29.3% and 50.6% of respondents had at least 1 school-age child in their household, 76.8% and 74.4% of respondents had received at least 1 dose of the COVID-19 vaccine, 23.7% and 26.0% of respondents were living with a chronic condition that put them at higher risk of COVID-19 related complications, 32.0% and 34.4% of respondents had regular contact with a clinically vulnerable individual, and 7.0% and 9.6% of respondent were pregnant. With respect to the political affiliation, under the “vaccine” survey scenario, 40.0%, 26.3%, and 31.0% of participants identified as Democratic, Independent, and Republican, respectively, and under the “no vaccine” survey scenario, 36.8%, 24.4%, and 36.0% of participants identified as Democratic, Independent, and Republican, respectively. The distributions of other socioeconomic characteristics among our survey participants are summarized in Supplementary Table S3.

**Figure 1:**
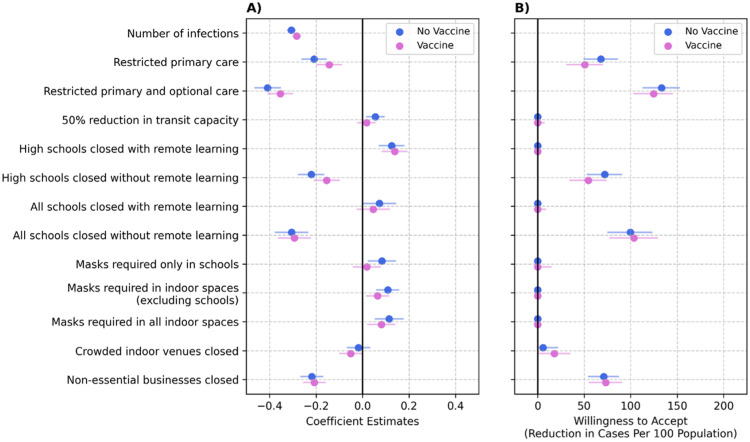
Coefficient estimates (panel A) and willingness to accept (WTA) (panel B) for both versions of the survey (where vaccines were or were not assumed to be available). WTA is the minimum reduction in the number of cases per 100 population during the next month that a nonpharmaceutical intervention (NPI) should achieve for a population to find the NPI worthwhile. WTA = 0 implies that, on average, population members found the NPI acceptable, even with minimal impact on cases in the next month. The error bars show the 95% confidence intervals.

We estimated the average change in a population member’s utility due to changes in COVID-19–related outcomes or the use of each NPI over the next month. Our analysis suggests that, on average, our survey participants had a significant preference in favor of scenarios with a low infection rate during the next month, maintained access to primary and optional care, available remote learning if schools are closed, and keeping nonessential businesses (such as restaurants, bars, and gyms) open (Figure 1A). On average, our survey participants were indifferent about reducing transit capacity, closing all schools when a remote learning option is provided, mask mandates, and closing crowded indoor event venues (such as clubs and theaters) (Figure 1A). The availability of the vaccine did not affect the magnitude of these preferences (comparing blue and purple dots in Figure 1A).

On average, our survey participants found closing all school grades without remote learning and business closures acceptable only if they could achieve a reduction of >75 cases per 100 population within a month (Figure 1B). This WTA threshold was smaller, at about 50 cases per 100 population within a month, for closing high schools without offering remote learning. On average, our survey participants preferred the scenario with schools open when remote learning is unavailable, and with regular access to primary and optional care independent of infection risk over the next month (Figure 1B).

**Figure 2:**
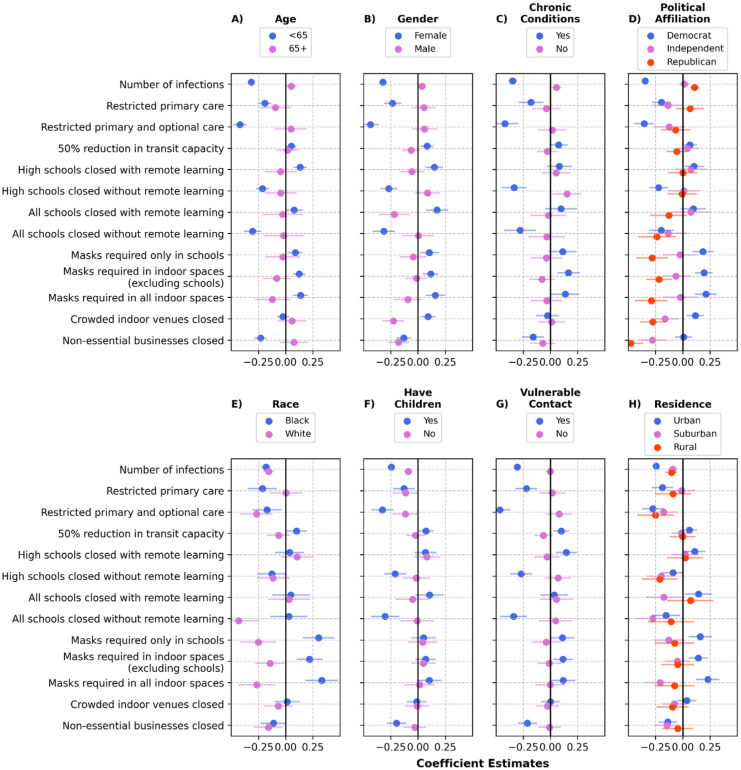
Coefficient estiamtes for different population subgroups characterized by age (panel A), gender (panel B), whether living with chronic conditions (panel C), political affiliation (panel D), race (panel E), whether living with school-aged children (panel D), whether having a clinically-vulnerable contact (panel G) and residence (panel H), under the “no vaccine” survey scenario.

**Figure 3:**
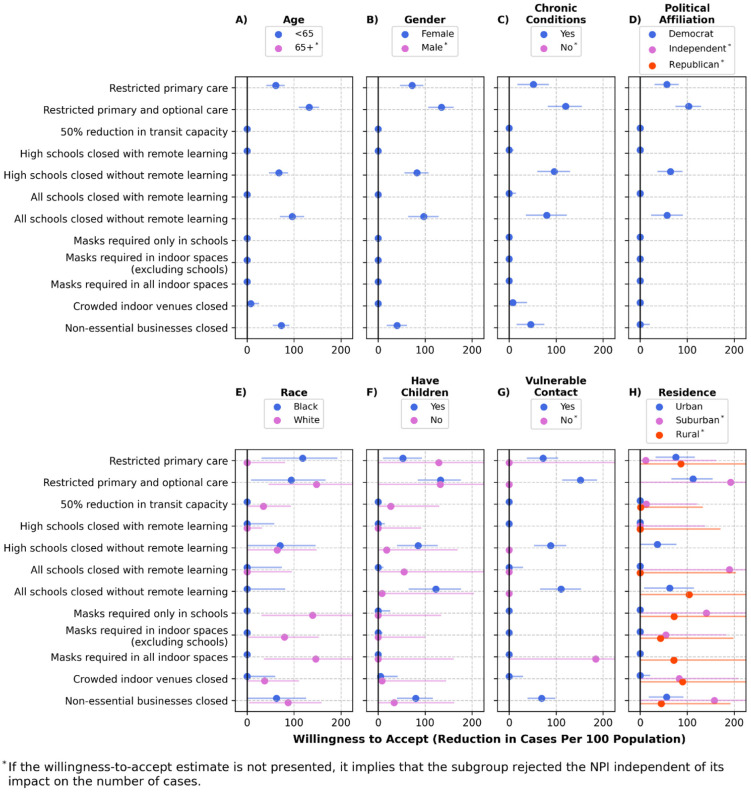
Willingness to accept (WTA) threshold for different nonpharmaceutical interventions (NPIs) among different population subgroups characterized by age (panel A), gender (panel B), whether living with chronic conditions (panel C), political affiliation (panel D), race (panel E), whether living with school-aged children (panel D), whether having a clinically-vulnerable contact (panel G) and residence (panel H), under the “no vaccine” survey scenario. WTA = 0 implies that, on average, population members found the NPI acceptable even with minimal impact on cases during the next month. If the WTA is not presented for a subgroup (e.g., for male in panel B), it implies that the subgroup rejected the NPI independent of its impact on the number of cases; this is because the change in utility due to increases in the number of cases was estimated to be positive for these subgroups (see Figure 2). See Supplementary Figure S3 for results under the “with vaccine” survey scenario.

Our analysis revealed substantial differences in WTA thresholds among different population subgroups. Increases in COVID-19 cases did not lower the utility of participants who were male, older than 65 y, without chronic conditions, Independent, or Republican (Figure 2A–D); hence, these groups rejected all NPIs considered here (Figure 3A–D). The utility of these subgroups was also not meaningfully affected by restricted access to primary and/or optional care (Figure 2A–D). Compared with men, women had a stronger preference to minimize the number of COVID-19 cases and to maintain access to primary and optional care. Women strongly opposed closing schools without offering remote learning but found mask mandates and closing crowded indoor venues acceptable at any level of effectiveness (WTA = 0 cases per 100 population). Compared with Independents and Republicans, Democrats had a stronger preference to minimize the number of COVID-19 cases and maintain access to primary and optional care (Figure 2D). In contrast to Independents and Republicans who rejected all NPIs, Democrats were willing to accept all forms of NPIs regardless of their effectiveness; the only exception was school closure without remote learning, for which Democrats’ WTA threshold was about 50 cases per 100 population within a month (Figure 3D). Black and White participants had similar preferences with respect to COVID-19 cases, access to primary and optional care, school closure with remote learning, and business closures. However, compared with White participants, Black participants were substantially more receptive to mask mandate policies (Figures 2E and 3E).

Females, Black participants, Democrats, participants who were younger than 65, lived with chronic conditions, had children, were in regular contact with individuals at risk of COVID-19 complications, or lived in urban areas were, in general, more receptive to NPIs. These subgroups found reductions in public transit capacity, school closures with remote learning, mask mandates, and the closure of crowded indoor venues acceptable, even if these NPIs have minimal impact on the number of cases. Despite being more receptive to NPIs, these subgroups too revealed high WTA thresholds (> 50 cases per 100 population within a month) for school closures without remote learning (except Black participants) and for closing nonessential businesses (except Democrats) (Figure 3). Similar patterns of preferences were observed under both survey scenarios of vaccine availability (Figure S2 and Supplementary Figure S3).

## Discussion

While NPIs, such as mask mandates and school and business closures, were critical tools for controlling the spread of SARS-CoV-2, they faced objections from many politicians and citizens in the United States. To quantify individuals’ preferences for the use of various NPIs in the United States, we conducted DCEs using data from a US population sample. Our analysis identified the number of COVID-19 cases over the next month, access to primary and optional care, the availability of remote learning options when schools are closed, and access to nonessential businesses (such as restaurants, bars, and gyms) as the most important attributes influencing population preferences. On average, the preferences of our survey participants were not significantly affected by reductions in public transit capacity, school closures when remote learning was offered, mask mandates, or the closure of crowded indoor venues (such as theatres and clubs). These preferences were not affected meaningfully by the availability of an effective vaccine.

The lack of access to primary and optional care and the closure of all school grades without a remote learning option significantly reduced the population’s utility. On average, our survey participants rejected scenarios with these control measures in place, regardless of their impact on infection risk (Figure 1B). Restricting primary care, closing high schools without remote learning, and closing nonessential businesses were among the second most important attributes; on average, our survey participants were willing to accept an increase of >50 COVID-19 cases per 100 population to avoid scenarios with these interventions in place (Figure 1B). While closing schools and nonessential businesses could reduce the transmission of SARS-CoV-2, there is no evidence to suggest that each of these interventions could reduce the number of COVID-19 cases by 50 cases per 100 population within a month.^1,42–46^ This survey suggests that our participants did not consider it appropriate to use these interventions to reduce COVID-19 cases.

On the other hand, our survey participants found indoor mask mandates and school closures with remote learning acceptable, even if they do not reduce cases (Figure 1B). Given the mounting evidence on the effectiveness of mask wearing, mask mandates, and school closures in reducing the transmission of respiratory viruses,^42,47–53^ our findings underscore the importance of providing high-quality masks and remote-learning infrastructure for pandemic preparedness and response.

Our survey participants strongly preferred maintaining uninterrupted access to primary and optional care. Lockdown policies implemented early in the COVID-19 pandemic were intended to prevent overwhelming health care systems and ensure access to care. However, the hospital capacity varies substantially across the US states and counties. In 2022, the number of hospital beds in the United States was 2.35 per 1,000 population, but varied between 1.6 and 5.0 per 1,000 population across states^54^ and more widely across counties, with higher densities typically found in urban centers and lower densities in rural areas.^55^ This highlights the importance of basing NPI use on local hospital occupancy and prioritizing control measures that can reduce COVID-19–related hospitalizations without closing schools and businesses.

Our analysis revealed substantial differences in the disutility associated with pandemic outcomes (e.g., COVID-19 infections) and NPIs across a range of socioeconomic and demographic groups. The utility of males, individuals older than 65 y, healthy individuals with no chronic conditions, Republicans, and Independents was not affected considerably by the number of COVID-19 cases or the limited access to primary care (Figure 2). These groups rejected all NPIs considered here (Figure 3). This is consistent with previous studies that have shown significant differences in attitudes toward COVID-19 and public health measures across genders and political groups. Women and Democrats are more likely to view COVID-19 as a serious health problem and agree to comply with public health measures.^56,57^ While older adults were generally cautious and tended to follow the health guidelines during the pandemic, they faced challenges due to disruption to daily routines, irregular access to food and medication, and increased feelings of loneliness and isolation.^58,59^ Our analysis suggests that for older adults, the disutility caused by NPIs outweighed their benefits in reducing COVID-19 cases.

This study has several limitations. First, temporal distancing may have affected participants’ recollection of their experiences with NPIs, as many of these measures ended at least 2 y before the survey was disseminated.^60,61^ Second, as with any stated-preference studies, our analysis relied on individuals selecting between hypothetical scenarios. Individuals may struggle to predict and evaluate future events accurately or tend to believe they are less likely to experience adverse outcomes, which could have influenced how participants responded to questions about hypothetical scenarios.^62,63^ Third, we use the Qualtrics internet panel to recruit participants for our DCEs. Although quotas improve demographic representativeness on observable characteristics (like age, gender, race, and income; Supplementary Table S1), the findings may not be fully generalizable to the broader US adult population, as the panel comprises individuals with internet access who are willing to participate in online surveys. This may underrepresent groups with limited internet access or those less inclined to participate in online research. However, we note that in 2024, 96% of US adults reported using the internet at least occasionally, and this percentage did not vary substantially across age, race and ethnicity, gender, income, and education groups.^64^ Fourth, our survey samples included a substantial number of participants living with school-aged children (Supplementary Table S3), which is essential for accounting for the impact of living with school-aged children on participants’ preferences for school closure strategies. However, we were unable to include this as a sampling quota since this information was not available for participants in the Qualtrics internet panel.

The fifth limitation arises from the heterogeneity in NPI implementation across states. While we attempted to standardize participants’ understanding of each intervention at the outset of the survey, their personal experiences may still have shaped their perceptions, leading to response variability. Sixth, the politicization of the pandemic is another factor that may have introduced bias, as political identity likely influenced how participants viewed NPIs rather than how NPIs actually affected their utility.^38,65^ Seventh, perceptions of risk and infection severity could influence a participant’s preference for NPI use. Although we asked participants whether they had any chronic conditions that would put them at a higher risk of serious illness from COVID-19, we did not explicitly collect their assessment of risk and infection severity. Eighth, we used subgroup analyses to assess how participants’ preferences vary by select sociodemographic characteristics (Supplementary Figure S2 and S3). However, many sociodemographic characteristics are correlated, and subgroup analyses alone do not isolate the unique contribution of each factor, which can be investigated by including interactions between pandemic attributes and all sociodemographic variables simultaneously. Estimating a fully interacted mixed logit model with all sociodemographic characteristics poses challenges of model complexity, convergence, and interpretability. We therefore estimated a reduced interaction model based on the subgroup analysis presented in Supplementary Figure S2 and S3. Finally, the context dependence of values and preferences might limit the generalizability of our findings beyond the US population. However, other DCE studies also suggested the general willingness in the United States,^22,66^ United Kingdom,^24,67^ Netherlands,^68^ France,^23,25^ and Australia^69^ to trade off pandemic-related health outcomes to relax NPIs.

The standard approach to resource allocation in health care relies on cost-effectiveness analysis (CEA).^70,71^ However, the application of CEA to guide NPI use is limited. CEA requires estimates of the long-term financial and health consequences of alternatives under consideration, which are not available for NPIs, especially for economically and socially marginalized groups. The DCEs described here could provide a fast, community-engaged alternative for informing public health measures during pandemics. The WTA estimates from this approach could be used to determine the reduction in cases required to justify the use of each NPI at a specific level. Furthermore, understanding individuals’ preferences within a population regarding different NPIs is essential for developing effective, likely to be accepted, and adhered-to mitigation strategies.

## Supplemental Material

sj-docx-1-mpp-10.1177_23814683261455894 – Supplemental material for Acceptability of Nonpharmaceutical Interventions to Prevent the Risk of COVID-19 Infection in the United States Supplemental material, sj-docx-1-mpp-10.1177_23814683261455894 for Acceptability of Nonpharmaceutical Interventions to Prevent the Risk of COVID-19 Infection in the United States by Rachel E. Murray-Watson, Marcy Ekanayake-Weber, Ted Cohen, Janel Hanmer and Reza Yaesoubi in MDM Policy & Practice
