# Supplementary material for: Acceptability of Nonpharmaceutical Interventions to Prevent the Risk of COVID-19 Infection in the United States
Source: MDM Policy Pract. 2026 Jun 24;11(1):23814683261455894. doi: 10.1177/23814683261455894 (PMC13305725; doi:10.1177/23814683261455894)
Supplement: sj-docx-1-mpp-10.1177_23814683261455894 – Supplemental material for Acceptability of Nonpharmaceutical Interventions to Prevent the Risk of COVID-19 Infection in the United States [file sj-docx-1-mpp-10.1177_23814683261455894.docx]

**Supplementary Materials**

1. Additional analysis details
   1. Number of blocks

The Qualtrics Conjoint software utilizes the following formula to determine the number of blocks ^1^:

$$\text{Number of Blocks= }\frac{\text{Base Number × Maximum number of levels in any attribute}}{\text{Number of choices per question }\text{×}\text{ Number of questions}}$$

Given that we have more than 10 levels across all attributes, we set the base number to 1,000 ^1^; the maximum number of levels is 5, and there are 10 choice tasks with two scenarios to select from. This gives a result of 250 blocks.

- 1. Linear utility function

We specified a linear utility function, $U$, for the individual $n$ choosing scenario $j$ in the choice task $t$ as:

$$U_{njt}=\alpha_{n}{Scenario}_{nt}$$

$$+\beta_{MASK,2,n}Masking_{2njt}+\beta_{MASK,3,n}Masking_{njt}+\beta_{MASK,4,n}Masking_{4njt}$$

$$+ \beta_{SC,2,n}SchoolClosure_{2njt} + \beta_{SC,3,n}SchoolClosure_{3njt}$$

$$+ \beta_{SC,4,n}SchoolClosure_{4njt} + \beta_{SC,5,n}SchoolClosure_{5njt}$$

$$+ \beta_{BC,2,n} BusinessClosure_{2njt}+ \beta_{BC,3,n} BusinessClosure_{3njt}+ \beta_{BC,4,n} BusinessClosure_{4njt}$$

$$+ \beta_{TR,2,n}TransitReduction_{2njt}$$

$$+ \beta_{INF,n}NumberInfected_{njt}$$

$$+ \beta_{HC,2,n}HealthCareRestriction_{2njt}+ \beta_{HC,3,n}HealthCareRestriction_{3njt}$$

$$+ \epsilon_{njt}$$

Here,

- ${Scenario}_{nj}$ takes 1 if the participant $n$ selects the scenario presented on the right panel in the choice task $t$ (see Fig. S1 for an illustration).
- $NumberInfected_{2njt}$ is a continuous variable representing the infection rate in the scenario $j$ and a choice task $t$ presented to the participant $n$.
- $HealthCareRestriction_{knjt},k\in\{2,3\}$ are binary variables and take 1 if the access to health care is restricted at the level 2 or 3 (defined in Table 1) in scenario $j$ and a choice task $t$ presented to the participant $n$.
- All other variables (e.g., $Masking_{knjt}$) are binary variables and take 1 if the corresponding NPI is implemented at the level $k$ (defined in Table 1) in scenario $j$ and a choice task $t$ presented to the participant $n$.
  1. Willingness to accept (WTA) calculations

For NPI $k$ that is implemented at level $l$, we calculated willingness to accept (WTA) as $\hat{\beta}_{kl}/\hat{\beta}_{I}$, where $\hat{\beta}_{kl}$ is the estimated main effect for NPI $k$ if implemented at level $l$ and $\hat{\beta}_{I}$ is the estimated main effect for infection rate during the next month ^2^. Here, $\hat{\beta}_{kl}$ is calculated as the mean of coefficient estimates for the level $l$ of NPI $k$ over participants; and $\hat{\beta}_{I}$ is calculated as the mean of the coefficients $\beta_{INF,n}$ over $n$.

We note that if an increase in the number of cases and the use of an NPI reduces the population utility (that is, if $\beta_{I}<0$ and $\beta_{kl}<0$), then WTA is defined as above. However, for the following two cases, WTA should be handled differently: 1) if $\beta_{I}\geq0$ and $\beta_{kl}<0$, meaning that the increase in cases did not negatively impact population utility, but was negatively affected by NPI $k$ at level $l$, then WTA is implicitly infinity (i.e., NPI $k$ at level $l$ does not improve the population utility regardless of its ability to reduce the number of cases); 2) if $\beta_{kl}>0$, meaning that NPI negatively impacted the population utility $k$ at level $l$, then WTA is implicitly 0 (i.e., the average population member would not mind NPI $k$ at level $l$ even if it does not reduce the number of cases.

To characterize the confidence interval for WTA estimates, we used the following bootstrap algorithm:

Let $\hat{W}_{kl}=\hat{\beta}_{kl}/\hat{\beta}_{I}$ be the estimated WTA for the attribute $k$ at level $l$.

Let $\sigma_{kl}$ and $\sigma_{I}$ be the standard error of the coefficient estimates $\hat{\beta}_{kl}$ and $\hat{\beta}_{I}$.

Repeat the following for $M=10,000$ times.

Set $X_{n}^{*}$ to a sample from $\mathcal{N}\left( \hat{\beta}_{kl}, \sigma_{kl} \right)$,

Set $Y_{n}^{*}$ to a sample from $\mathcal{N}(\hat{\beta}_{I}, \sigma_{I})$.

Let $d_{n}^{*} = X_{n}^{*}/Y_{n}^{*} - \hat{W}_{kl}$

Set $\delta_{\alpha/2}$to ($1-\alpha/2$)100^th^ percentile of $d_{1}^{*}, d_{2}^{*}, \ldots, d_{M}^{*}$.

Set $\delta_{1-\alpha/2}$ to ($\alpha/2$)100^th^ percentile of $d_{1}^{*}, d_{2}^{*}, \ldots, d_{M}^{*}$.

Return ${[\hat{W}}_{kl}-\delta_{\alpha/2}, \hat{W}_{kl}-\delta_{1-\alpha/2}]$.

1. Supplementary Tables

| Table S1: Quotas for sampling based on the US Census data ^3^. | |
| --- | --- |
| **Characteristic** | **Quota (%)** |
| Age: 18-24 years | 13.55 |
| Age: 25-34 years | 17.96 |
| Age: 35-49 years | 23.51 |
| Age: 50-64 years | 22.02 |
| Age: > 65 years | 22.04 |
| Gender: Male | 48 |
| Gender: Female | 52 |
| Gender: Non-binary | Natural fallout |
| Race: White | 75 |
| Race: Black and African American | 13 |
| Race: Asian or Pacific Islander | 6 |
| Race: Native American, American Indian, Alaskan Native, other | 6 |
| Hispanic: Yes | 18 |
| Household income: < $35, 000 | 25 |
| Household income: $35, 000 − 75, 000 | 25 |
| Household income: $75, 000 − 150, 000 | 25 |
| Household income: > $150, 000 | 25 |

Table S2: Socio-economic, demographic, and health status questions asked of survey participants

| **Attributes** | **Levels** |
| --- | --- |
| Which of the following best describes your gender? | - Female - Male - Non-binary - Prefer not to answer Other |
| Which of the following best describes your race? Multiselect | - Black or African American - Native Hawaiian or Pacific Islander - American Indian or Native American or Alaskan Native - Middle Eastern North African Chinese - Vietnamese - Filipino - Samoan - Asian Indian - Japanese - Korean - Chamoan - White - Prefer not to answer - Self-describe |
| Would you describe yourself as Hispanic, Latino, or of Spanish Origin? | - Yes - No |
| What is your age group? | - 18-24 - 25-34 - 35-49 - 50-64 - ≥ 65 |
| Combining all income sources, how much money does your household earn each year? | - < $35, 000 - $35,000-75,000 - $75, 000 − 150, 000 - > $150, 000 |
| Which of the following best describes your place of residence? | - Rural - Suburban - Urban |
| Did you have at least one child in grade school (K -12) between March 2020 and March 2022? | - Yes - No |
| Did you live in a nursing home or assisted living facility between March 2020 and March 2022? | - Yes - No |
| Which was your primary state of residence between March 2020 and March 2022? | Participants choose one of 50 States or Washington DC |
| Have you received at least one COVID-19 vaccination? | - Yes - No |
| Do you have any chronic conditions that would put you at a higher risk of serious illness from COVID-19? | - Yes - No - Prefer not to answer |
| Were you in regular and close contact with someone at high risk of complications from COVID-19, such as an elderly family member or someone with heart disease? | - Yes - No |
| Are you covered by any health insurance plans? | - Yes - No |
| Which of these do you use as your primary news source? | - Social media (Instagram, Facebook, X (Twitter), TikTok) - Print media (newspapers, journals) - TV (including cable) - Radio or podcasts - News apps or websites |
| Which of the following describes your political affiliation? | - Democrat - Republican - Independent - Other |
| Are you self-employed? | - Yes - No |
| Were you able to work from home or work remotely between March 2020 and March 2022? | - Yes - No - Not applicable (studying, re- tired, not in paid employment) |
| Did you have access to a private vehicle to go to work, take your children to school, or run day-to-day errands between March 2020 and March 2022? | - Yes - No |
| What is the highest level of education that you have completed? | - High school or GED Some college credits - 1 or more years of college credit, no degree - Associate’s degree - Undergraduate degree - Postgraduate degree |
| Were you pregnant at any point between March 2020 and March 2022? | - Yes - No |

Table S3: Demographic and socioeconomic characteristics of study participants

| **Characteristic** | **No vaccine scenario** (N=2,519) | **Vaccine scenario** (N= 2,527) |
| --- | --- | --- |
| Gender |  |  |
| Female | 1304 (51.8) | 1336 (52.9) |
| Male | 1198 (47.6) | 1172 (46.4) |
| Non-binary | 10 (0.4) | 16 (0.6) |
| Self-define | 4 (0.2) | 2 (0.1) |
| Prefer not to answer | 3 (0.1) | 1 (0.04) |
| Race |  |  |
| American Indian or Native American or Alaskan Native | 21 (0.8) | 56 (2.2) |
| Asian/Asian American | 80 (3.2) | 72 (2.9) |
| Black or African American | 403 (16.0) | 345 (137) |
| White | 1793 (71.2) | 1870 (74) |
| Multiracial | 190 (7.6) | 58 (2.3) |
| Prefer not to answer | 29 (1.2) | 16 (0.6) |
| Hispanic, Latino, Spanish Origin |  |  |
| Yes | 444 (17.6) | 224 (17.5) |
| No | 2073 (82.3) | 2086 (82.6) |
| Age Group |  |  |
| 18-24 | 332 (13.2) | 299 (11.8) |
| 25-34 | 450 (17.9) | 465 (18.4) |
| 35-49 | 596 (23.7) | 618 (24.5) |
| 50-64 | 575 (22.8) | 589 (23.3) |
| *≥* 65 | 566 (22.5) | 556 (22.0) |
| Income ($) |  |  |
| *<* 35,000 | 613 (23.4) | 601 (23.8) |
| 35,000 - 75,000 | 646 (25.7) | 641 (25.4) |
| 75,000 - 150,000 | 813 (32.3) | 965 (38.2) |
| *>*150,000 | 447 (17.8) | 320 (12.7) |
| Geographic Region |  |  |
| Northeast | 474 (18.9) | 522 (20.7) |
| Midwest | 571 (22.8) | 631 (25.0) |
| South | 1117 (44.5) | 1146 (45.4) |
| West | 471 (18.7) | 422 (16.8) |
| Residence |  |  |
| Rural | 479 (19.0) | 476 (18.9) |
| Suburban | 1265 (50.2) | 1257 (49.7) |
| Urban | 775 (39.8) | 794 (31.4) |
| School-aged children in the household |  |  |
| Yes | 738 (29.3) | 1279 (50.6) |
| No | 1781 (70.7) | 1248 (49.4) |
| Living in a nursing home or assisted living |  |  |
| Yes | 118 (4.7) | 167 (6.6) |
| No | 2401 (95.3) | 2360 (93.4) |
| Vaccinated against COVID-19 |  |  |
| Yes | 1934 (76.8) | 1881 (74.4) |
| No | 585 (23.2) | 646 (25.6) |
| Living with a chronic condition |  |  |
| Yes | 597 (23.7) | 657 (26.0) |
| No | 1883 (74.8) | 1834 (72.6) |
| Prefer not to answer | 39 (1.6) | 36 (1.4) |
| Regular contact with a clinically vulnerable individual |  |  |
| Yes | 806 (32.0) | 869 (34.4) |
| No | 1713 (68.0) | 1658 (65.6) |
| Health insurance coverage |  |  |
| Yes | 2302 (91.4) | 2302 (91.1) |
| No | 217 (8.6) | 225 (8.9) |
| Primary news source |  |  |
| News apps or websites | 511 (20.3) | 443 (17.5) |
| Other | 34 (1.4) | 25 (1.0) |
| Print media | 89 (3.5) | 99 (3.9) |
| Radio or podcasts | 91 (3.6) | 102 (4.0) |
| Social media | 733 (29.1) | 784 (31.0) |
| TV | 974 (38.7) | 993 (39.3) |
| Do not read the news | 87 (3.5) | 81 (3.2) |
| Political affiliation |  |  |
| Democrat | 1008 (40.0) | 929 (36.8) |
| Independent | 663 (26.3) | 613 (24.3) |
| Republican | 780 (31.0) | 911 (36.0) |
| Other | 68 (2.7) | 74 (2.9) |
| Self-employed |  |  |
| Yes | 424 (16.8) | 435 (17.2) |
| No | 2095 (83.2) | 2092 (82.8) |
| Able to work remotely |  |  |
| Yes | 1040 (41.3) | 1103 (43.7) |
| No | 902 (35.9) | 907 (35.9) |
| NA | 577 (22.9) | 517 (20.5) |
| Had access to a private vehicle to commute to work |  |  |
| Yes | 1987 (78.9) | 2081 (82.3) |
| No | 532 (21.1) | 446 (17.7) |
| Highest Level of Education |  |  |
| High school or GED | 618 (24.5) | 660 (26.1) |
| Some college credits | 236 (9.4) | 226 (8.9) |
| *>*1 year college credits, no degree | 251 (10.0) | 245 (9.7) |
| Associate’s degree | 319 (12.7) | 352 (13.9) |
| Undergraduate degree | 611 (24.3) | 584 (23.0) |
| Postgraduate degree | 484 (19.2) | 462 (18.3) |
| Was pregnant during the pandemic |  |  |
| Yes | 176 (7.0) | 242 (9.6) |
| No | 1174 (93.0) | 2285 (90.4) |
|  |  |  |
|  |  |  |

Table S4: Icons illustrating each attribute level.

| **Attributes** | **Level** | **Icon** | **Citation** |
| --- | --- | --- | --- |
| **Mask Mandates** | No mask mandate in public settings. | 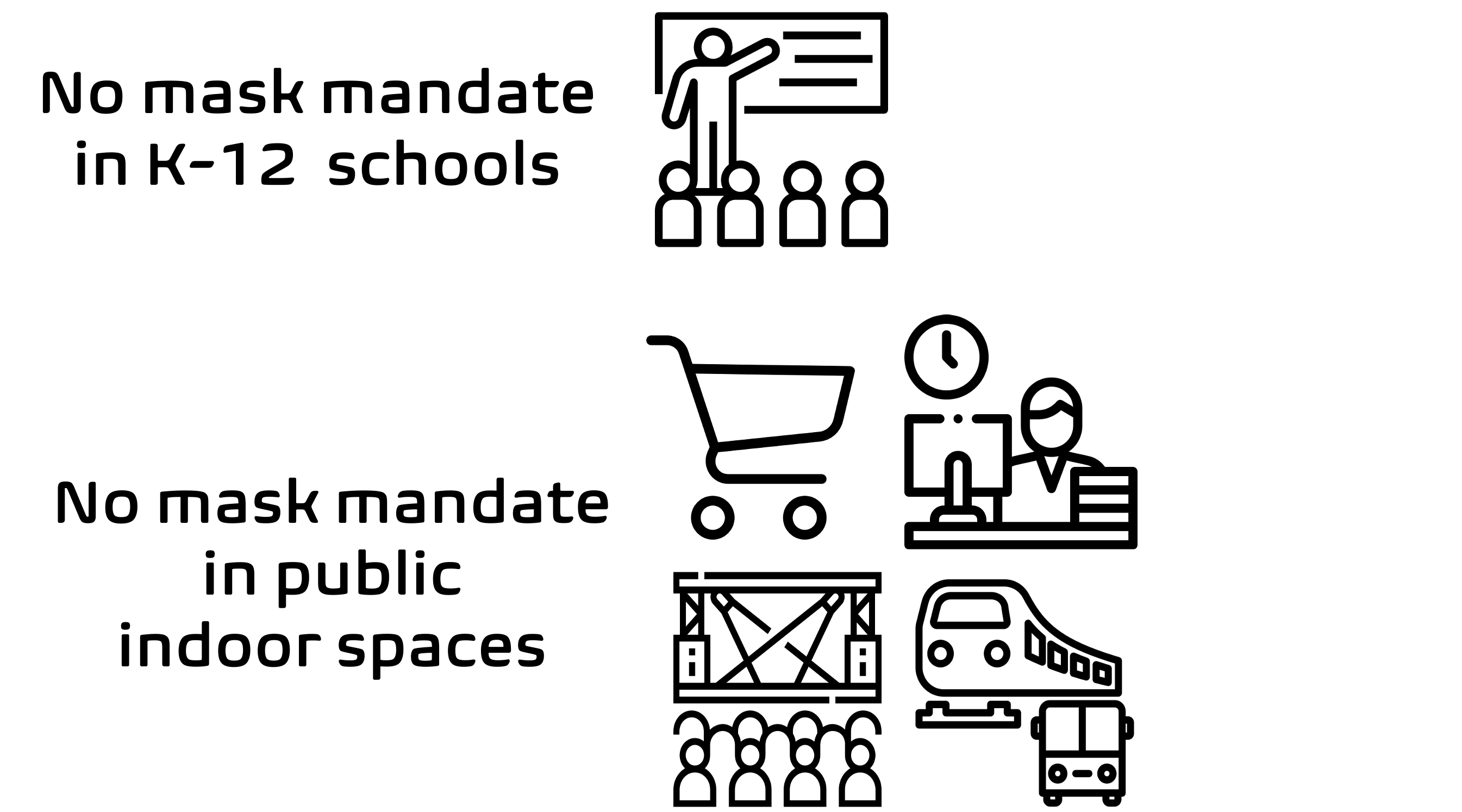 | ^4-6^ |
|  | Mask mandates in schools from kindergarten to 12th grade. | 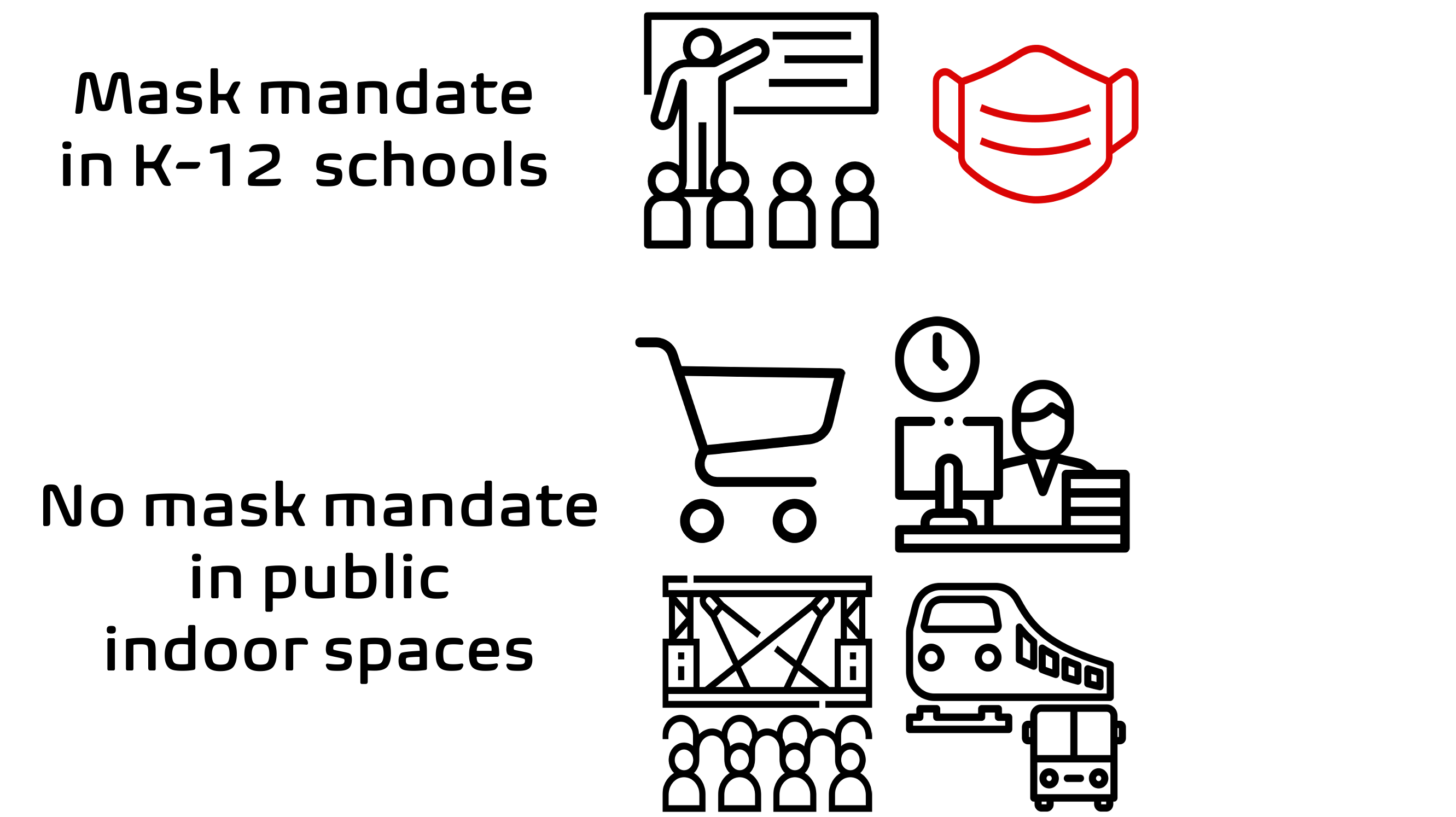 | ^4-6^ |
|  | Mask mandates in public indoor spaces such as shops, offices, cinemas theatres etc., but not in schools. | 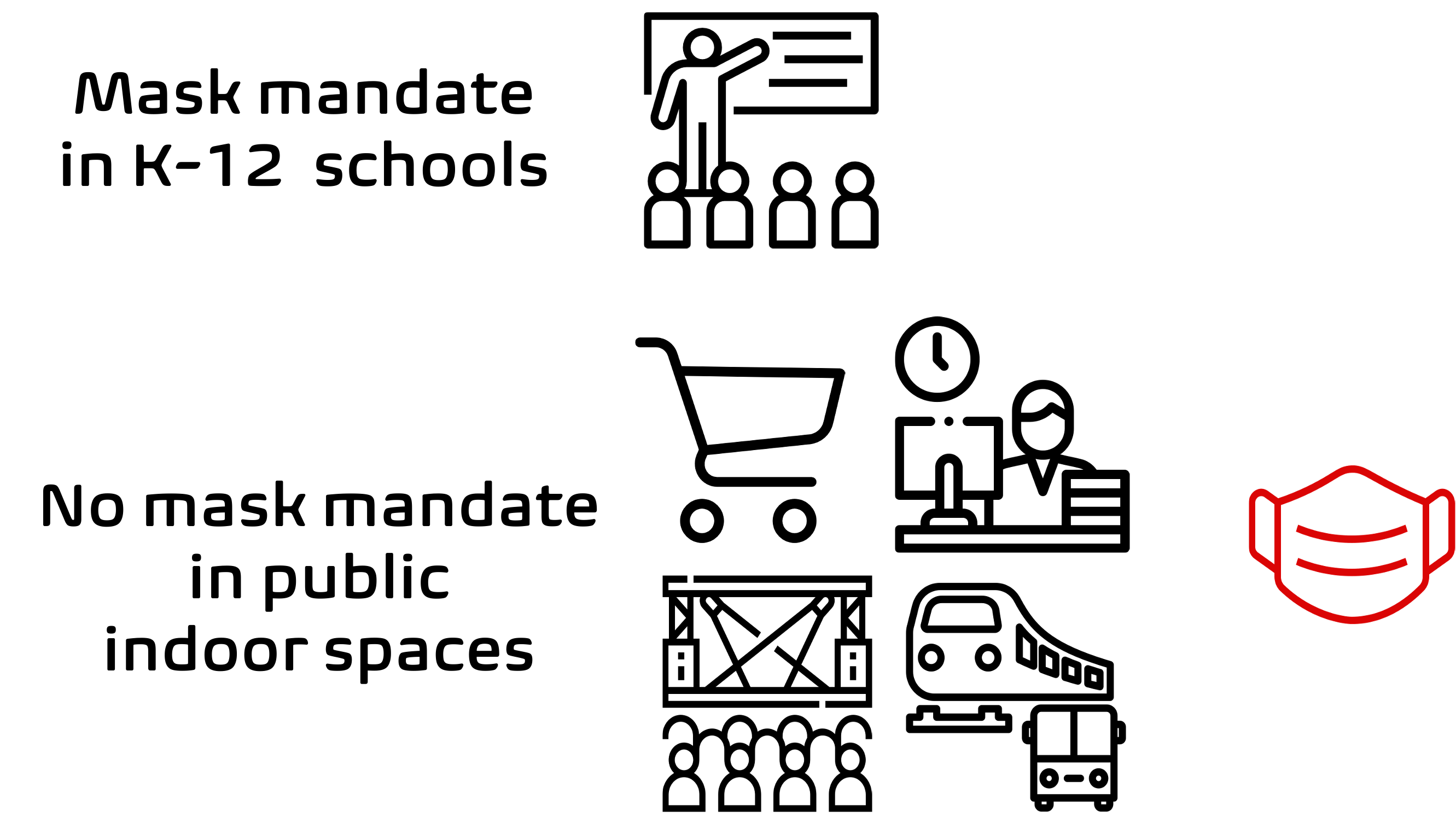 | ^4-6^ |
|  | Mask mandates in all public indoor spaces and schools from kindergarten to 12th grade. | 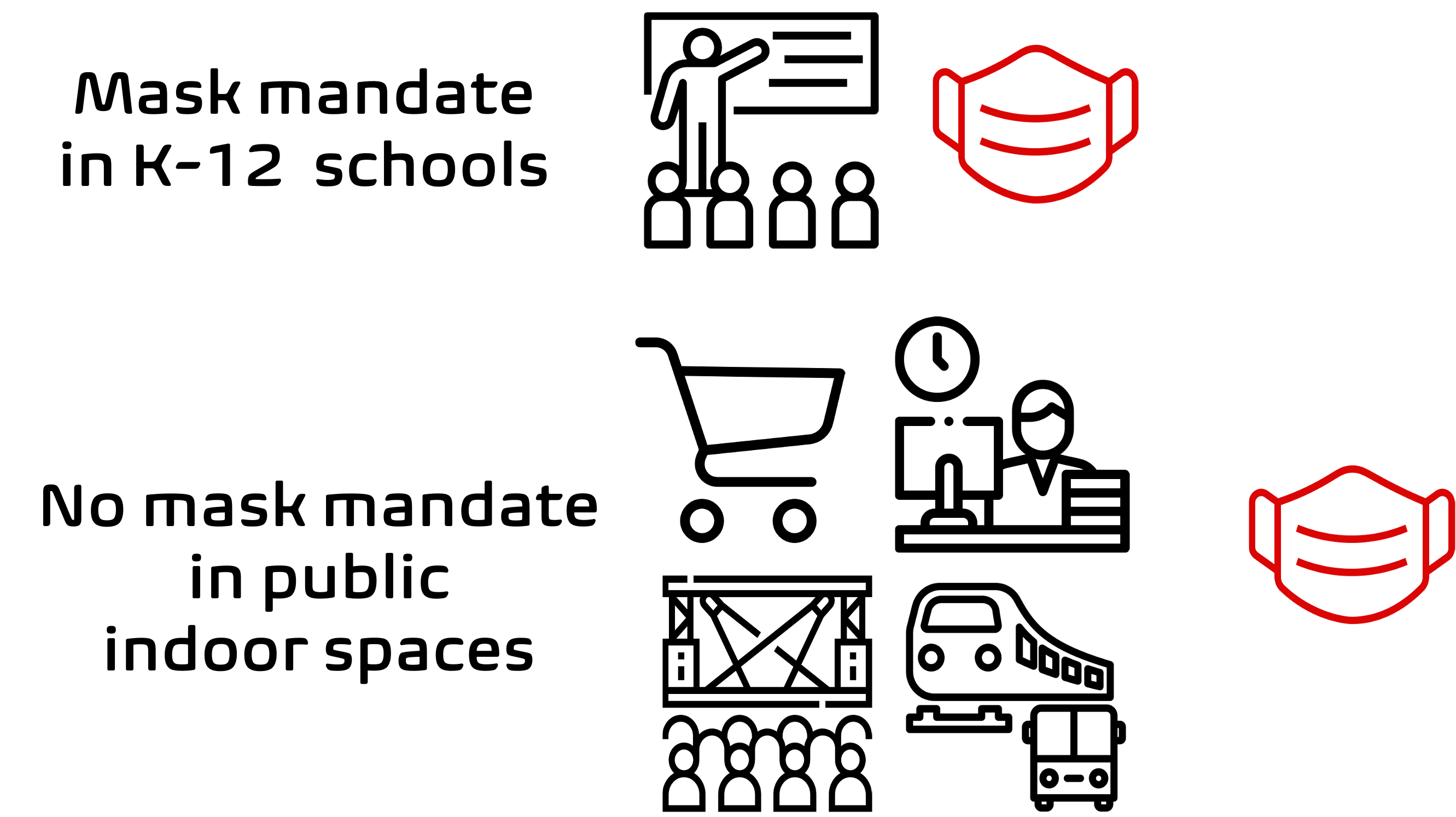 | ^4-6^ |
| **School Closures** | No schools close. | 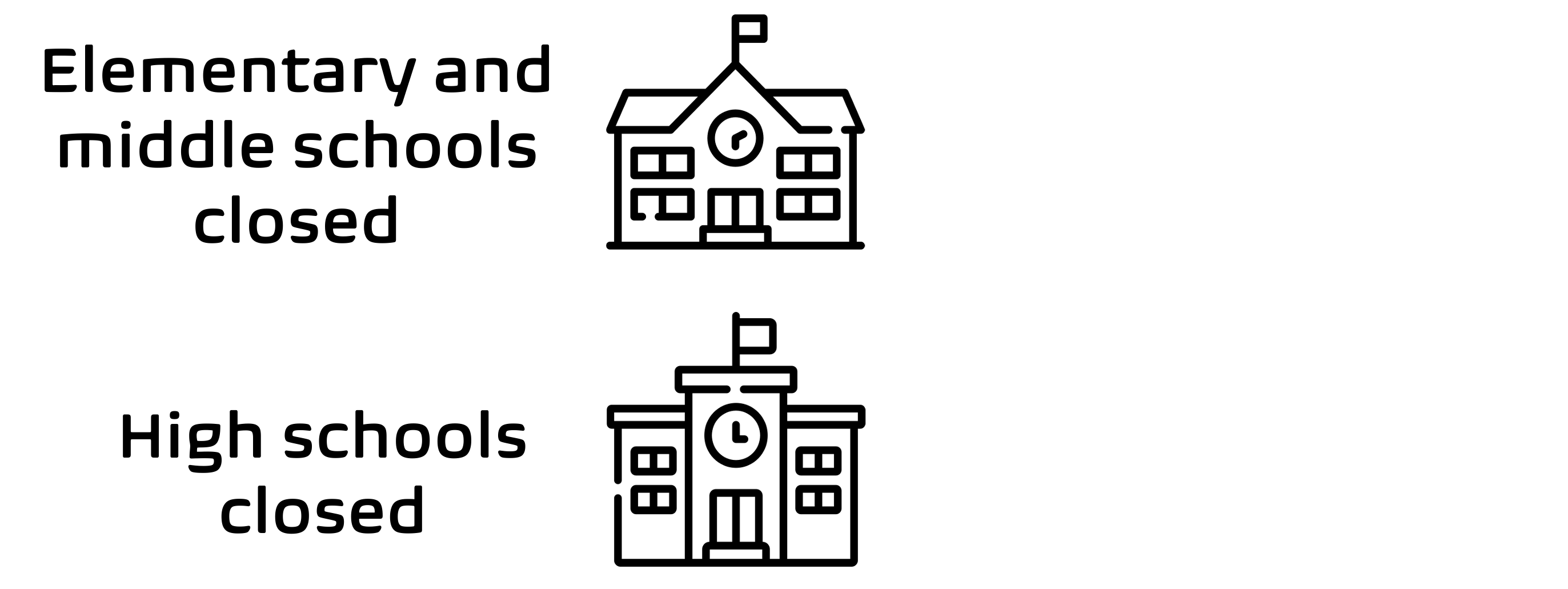 | ^7-9^ |
|  | Only high schools close, but remote learning is available. | 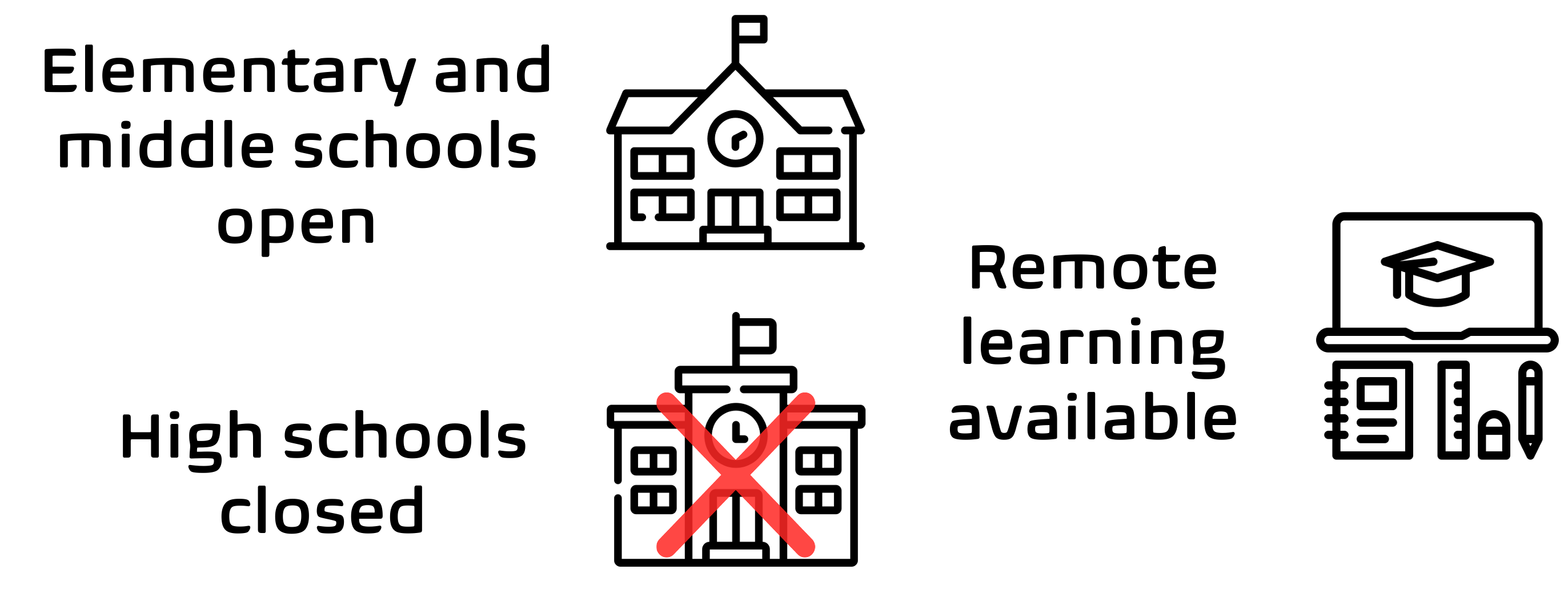 | ^7-9^ |
|  | Only high schools close, but remote learning is not available. | 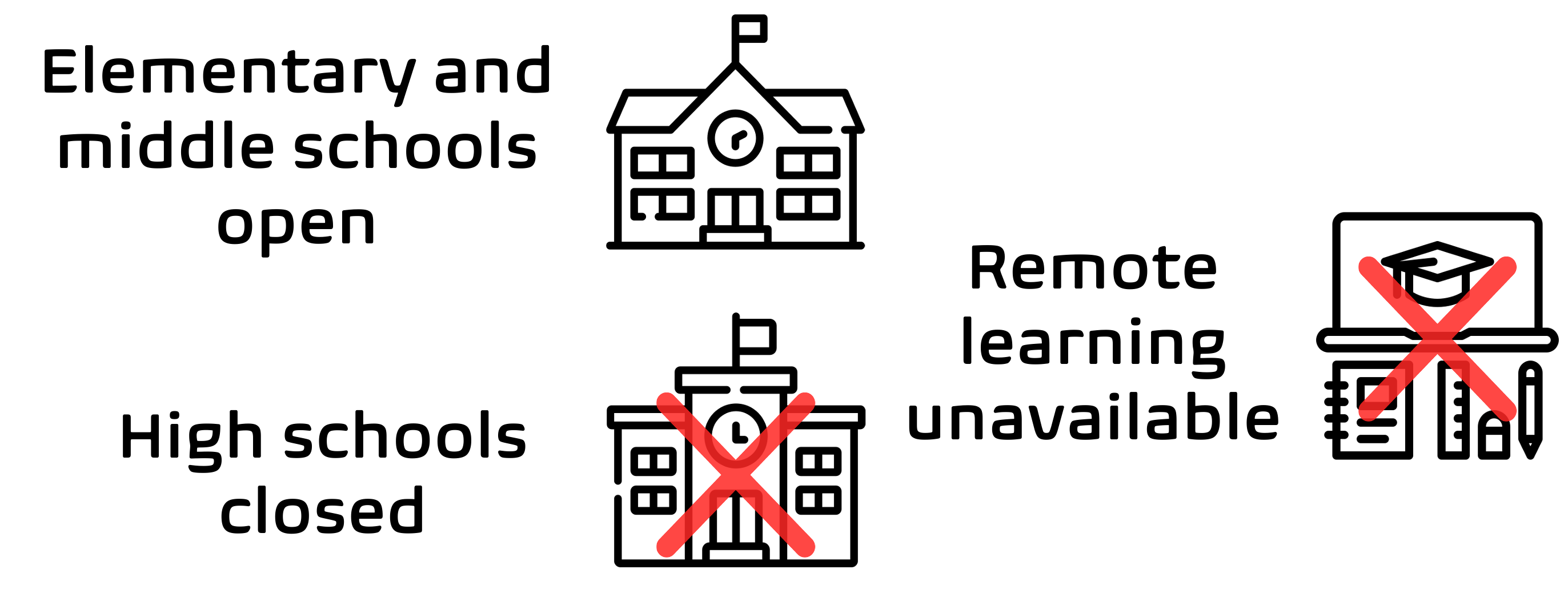 | ^7-9^ |
|  | All schools close but remote learning is available. | 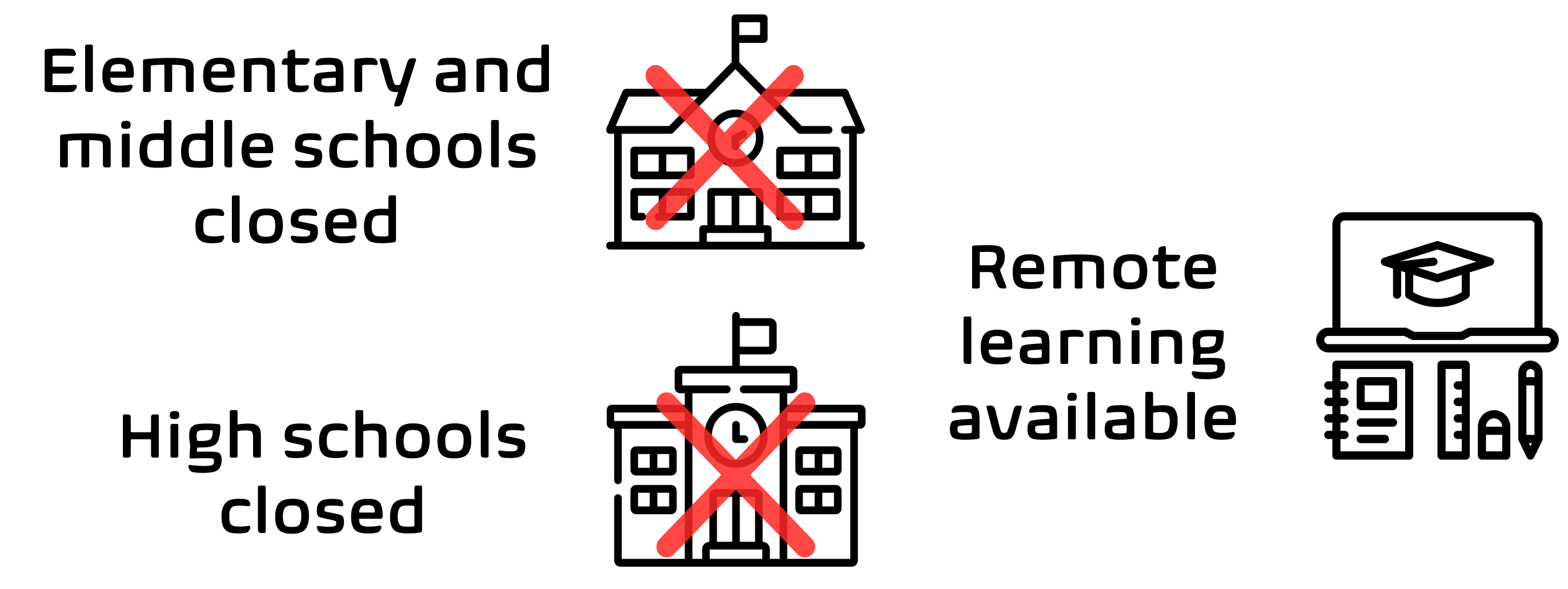 | ^7-9^ |
|  | All schools close, but remote learning is not available. | 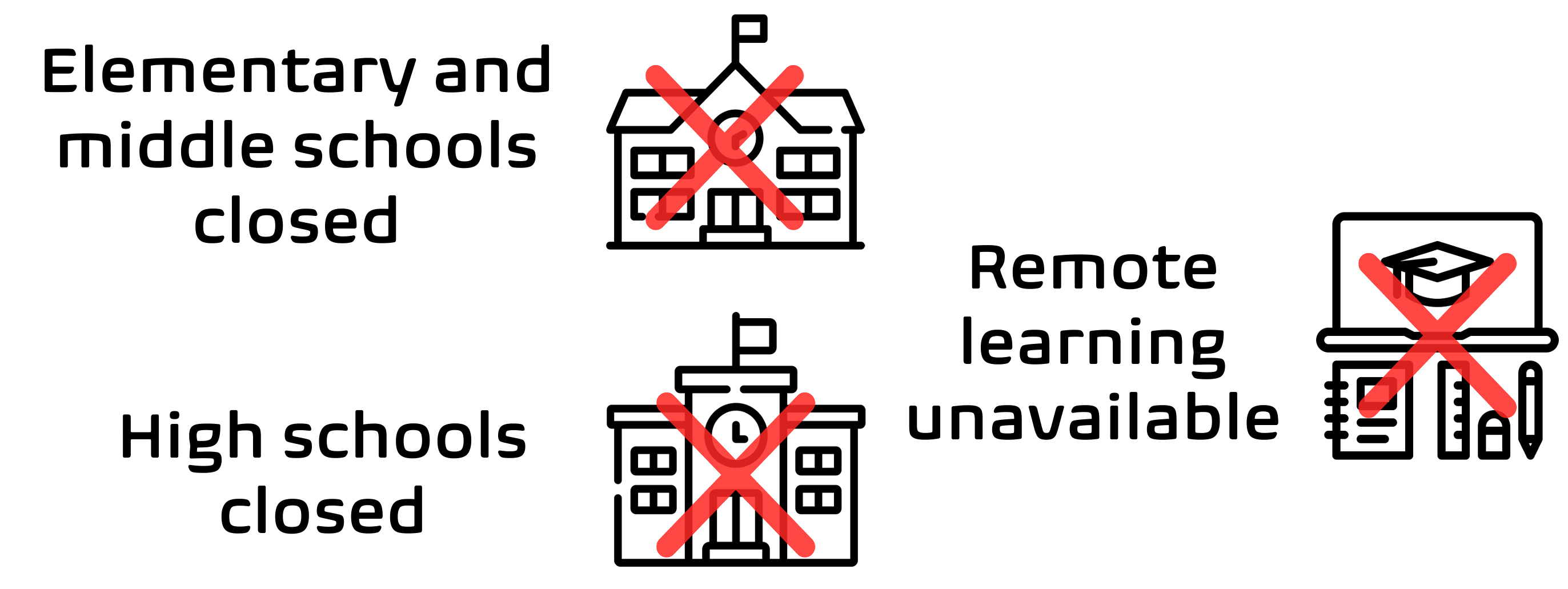 | ^7-9^ |
| **Business Closures** | No businesses close. | 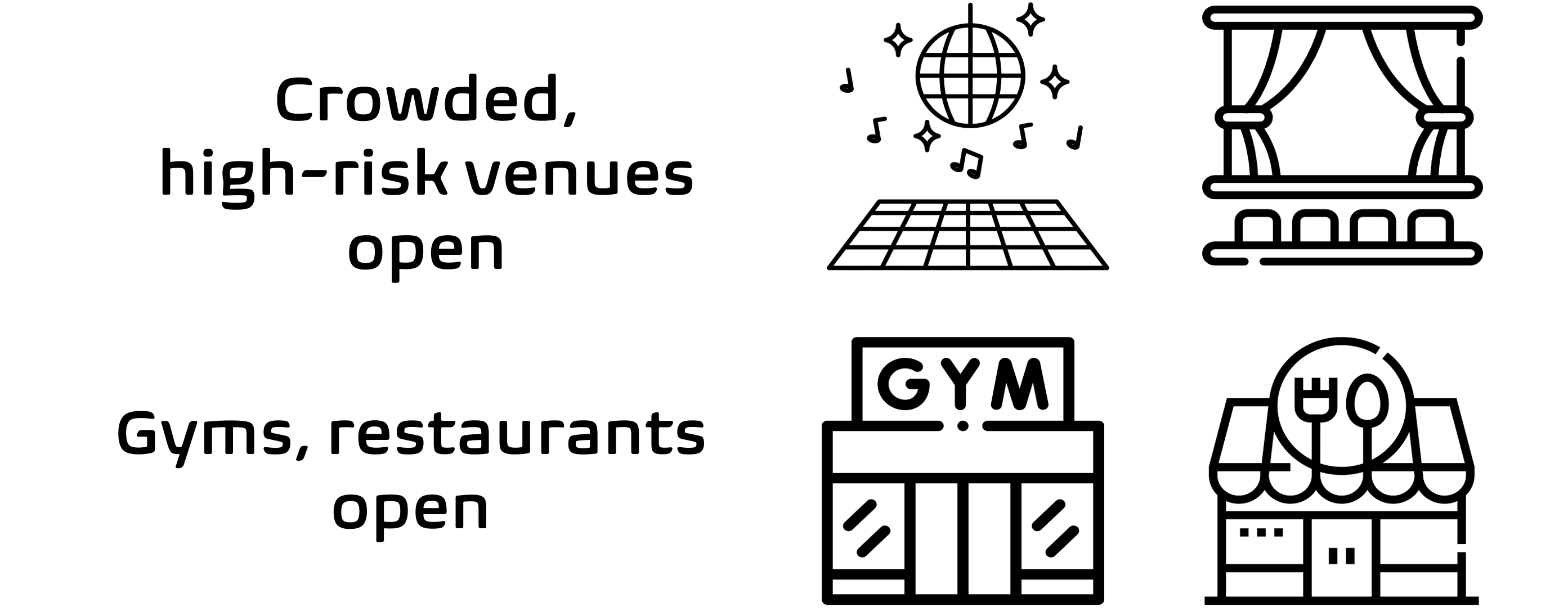 | ^10-13^ |
|  | Crowded indoor venues and event venues close. | 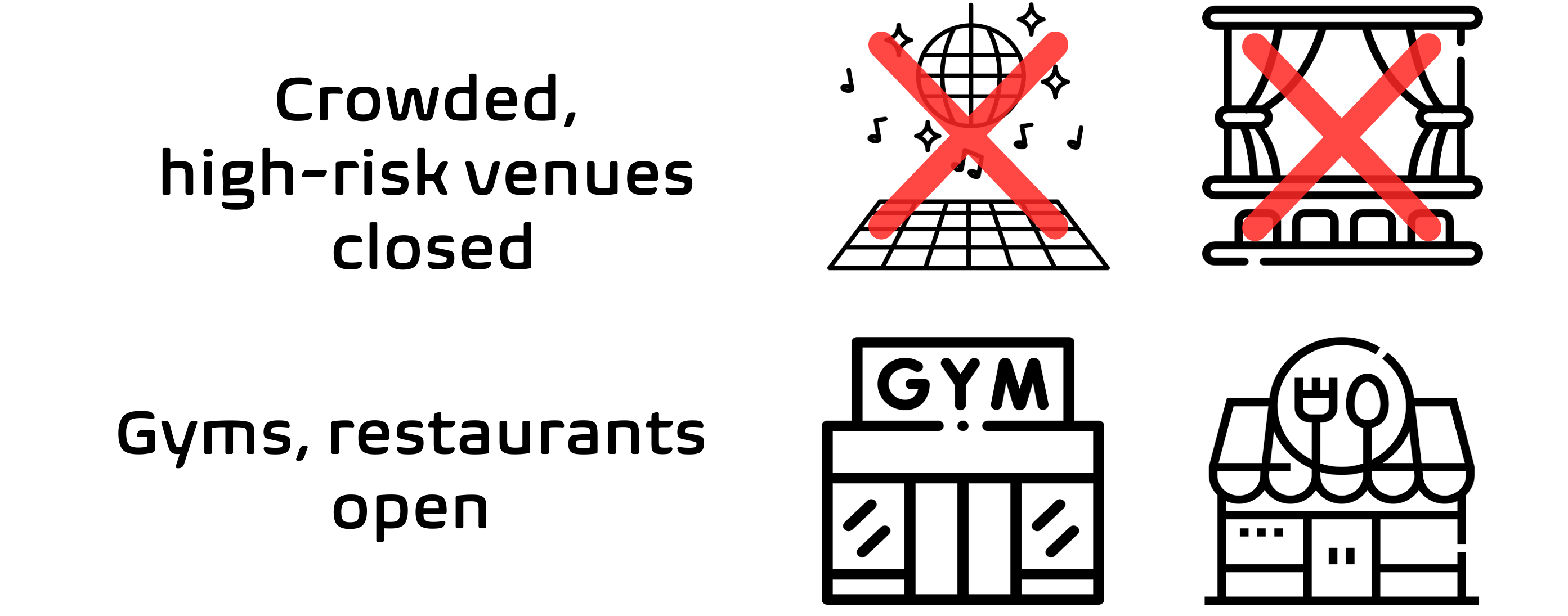 | ^10-13^ |
|  | All non-essential businesses close. | 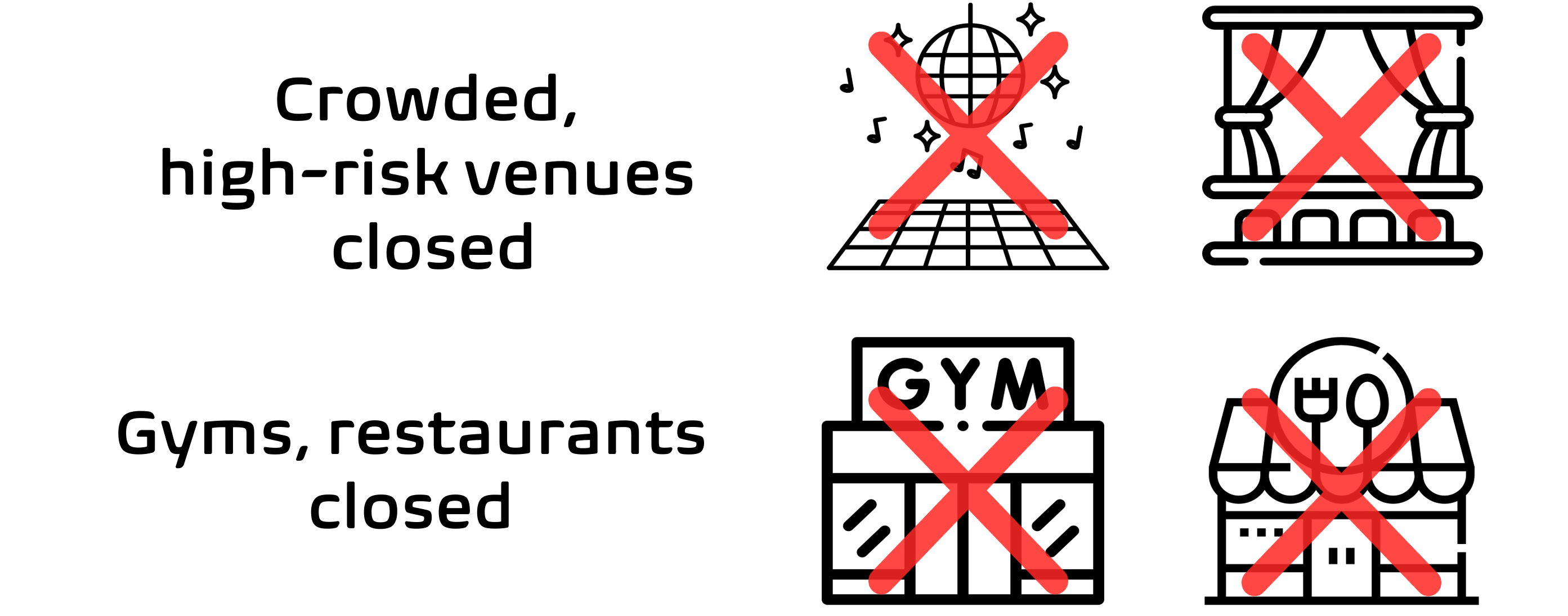 | ^10-13^ |
| **Reduction in Public Transit Capacity** | No reduction in public transit capacity. | 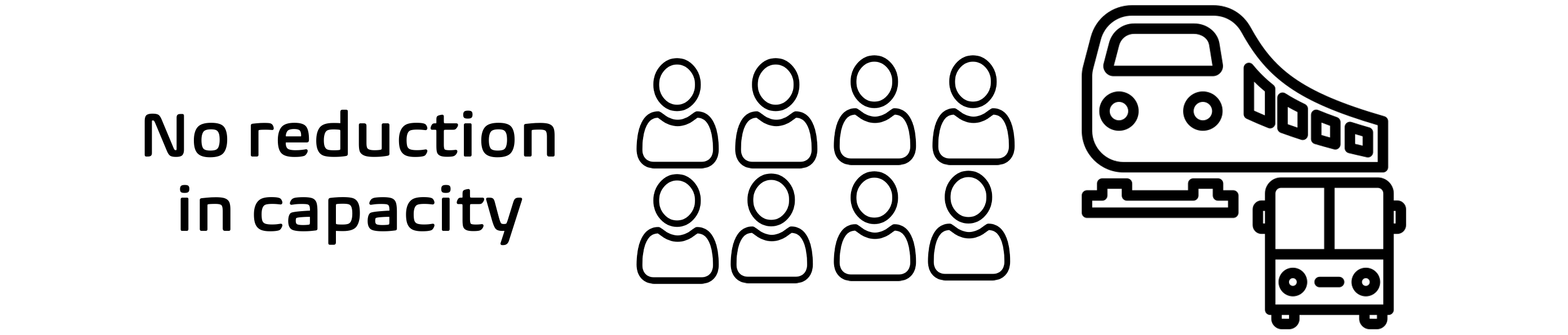 | ^14, 15^ |
|  | 50% reduction in public transit capacity. | 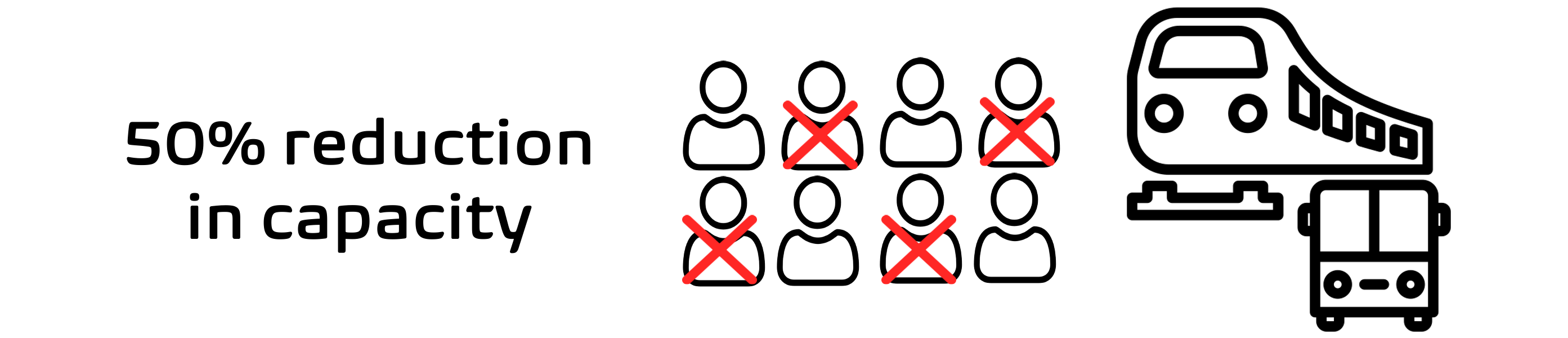 | ^14, 15^ |
| **Number of People Infected over the Next Month** | 1 in 100 people | 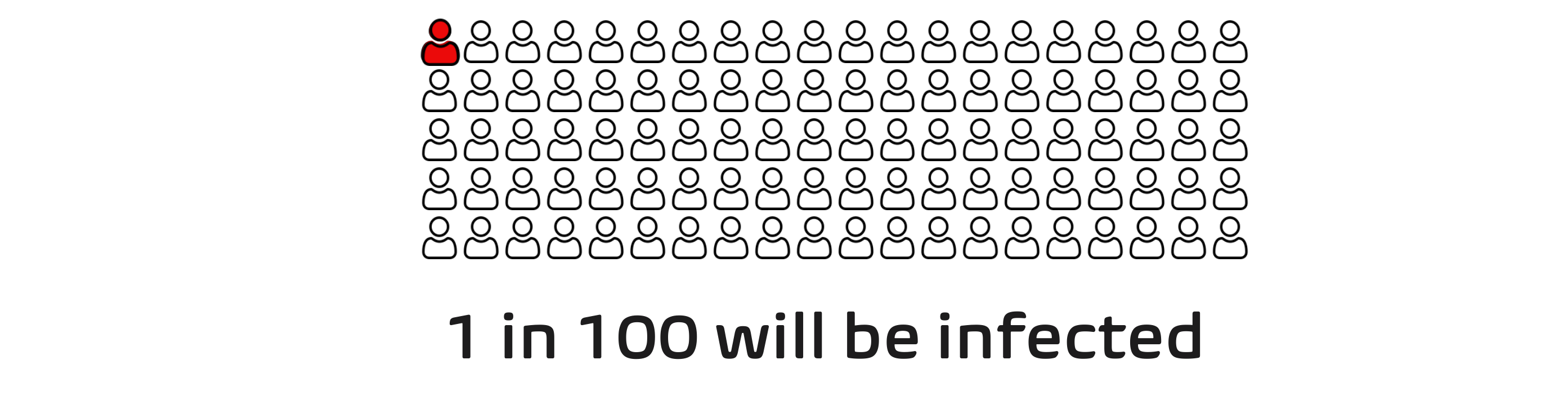 | ^14^ |
|  | 5 in 100 people | 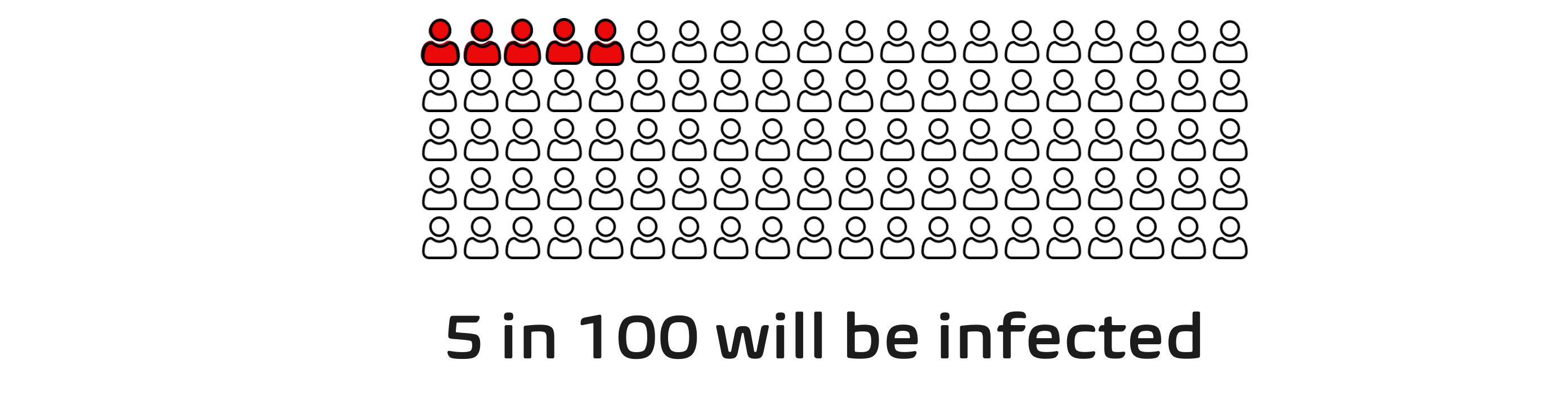 | ^14^ |
|  | 10 in 100 people | 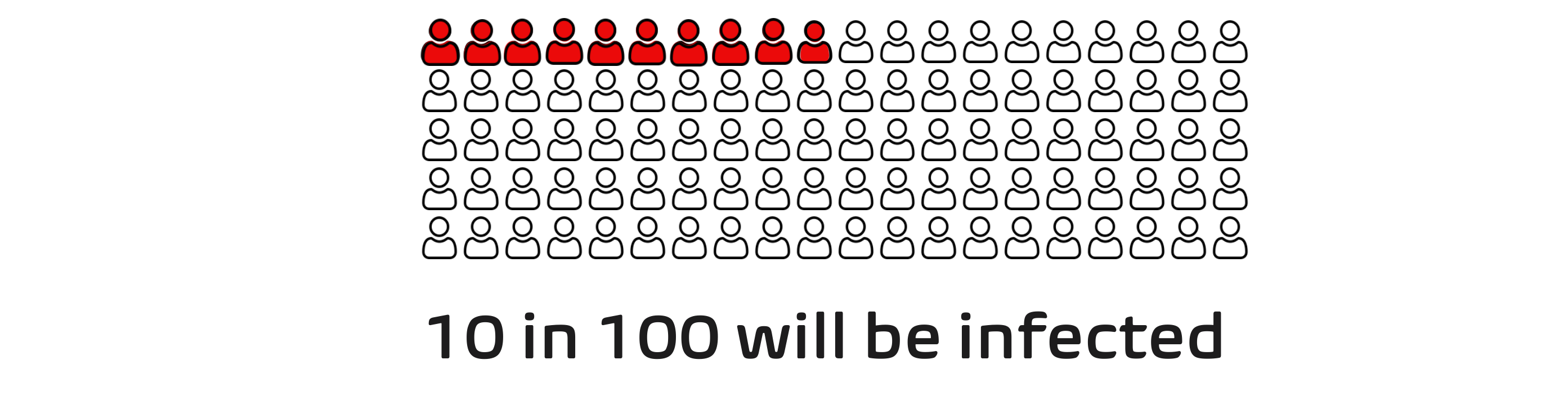 | ^14^ |
|  | 15 in 100 people | 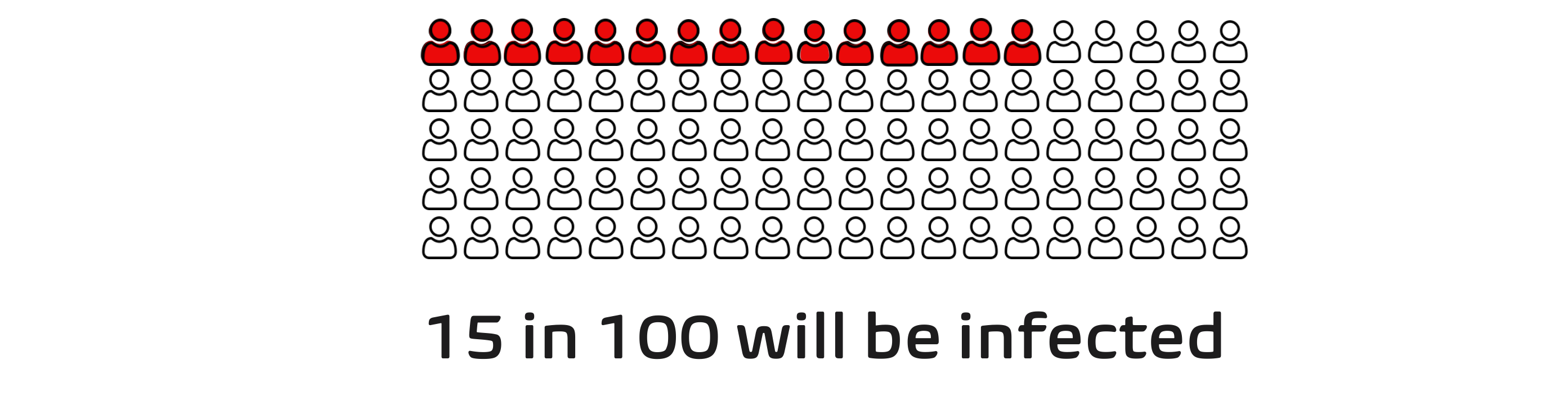 | ^14^ |
|  | 20 in 100 people | 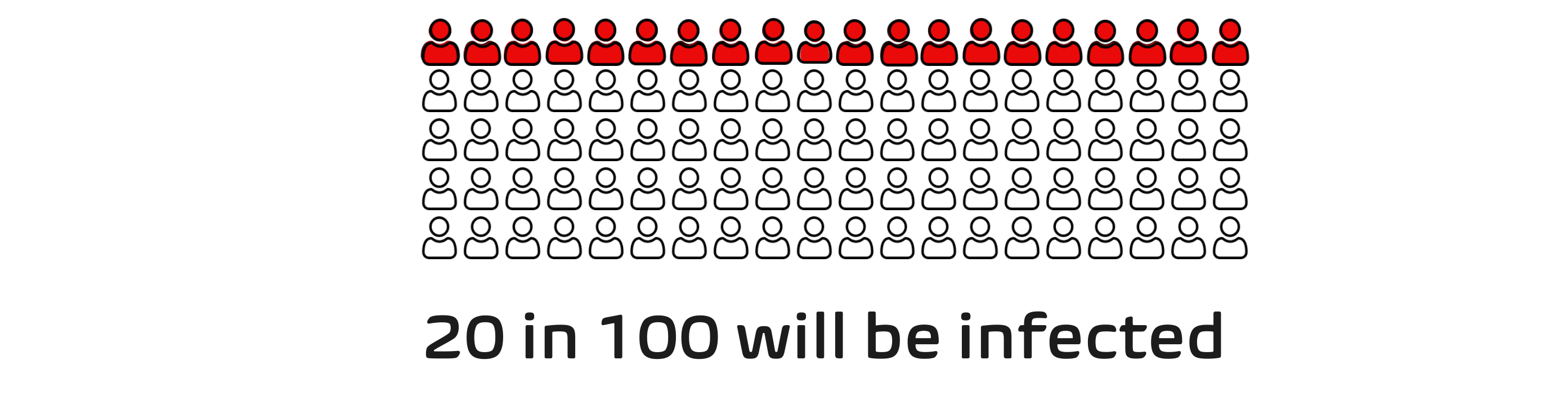 | ^14^ |
| **Access to Healthcare** | No restrictions. | 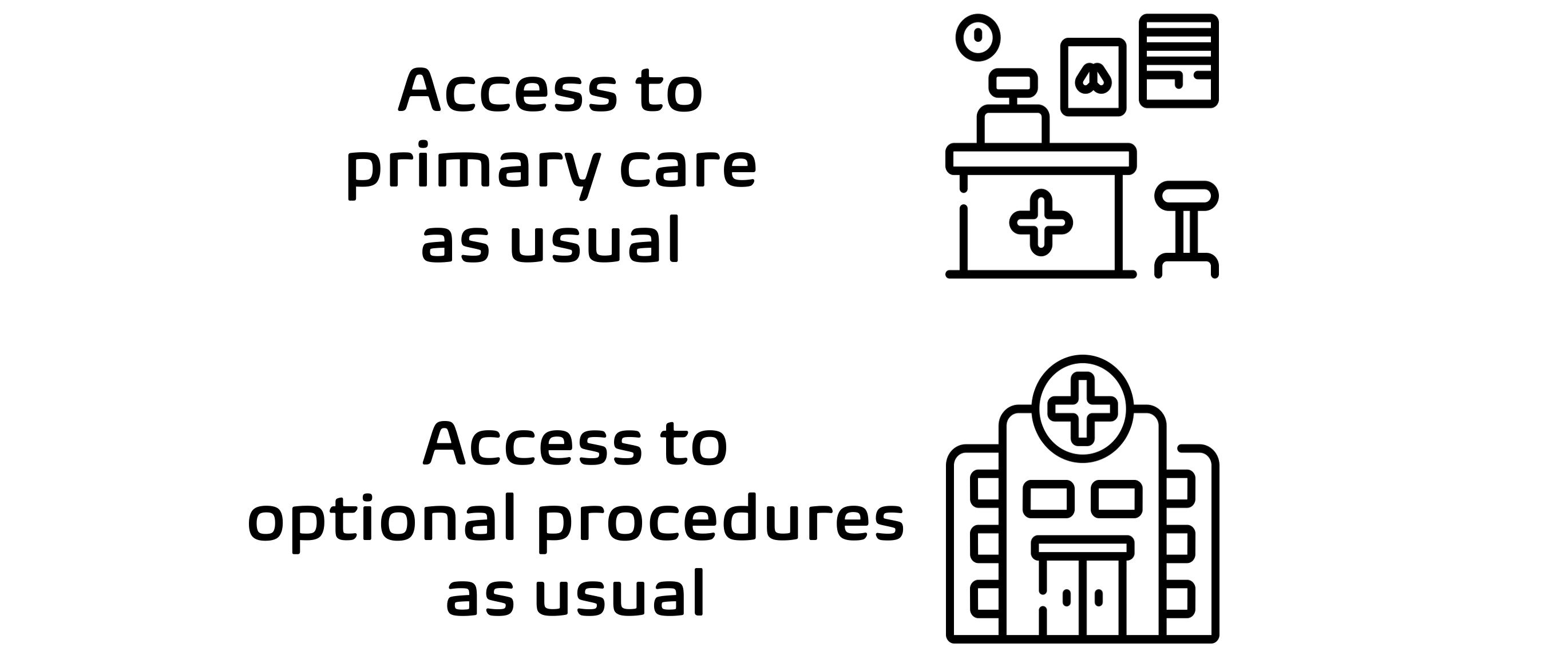 | ^16, 17^ |
|  | Access to primary care is limited (that is, routine care or preventative care, such as check-ups and treatment for non-urgent illnesses would be cancelled, delayed, or pro- vided by telemedicine). | 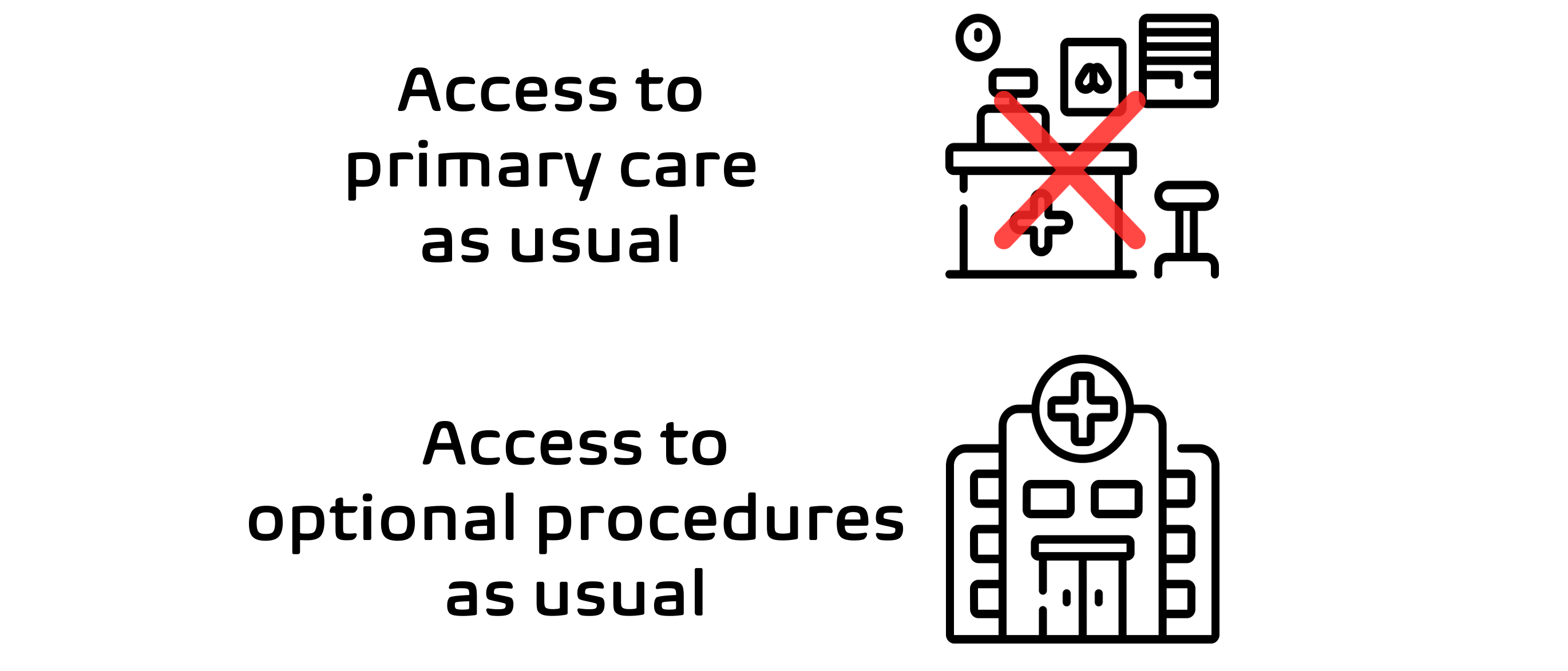 | ^16, 17^ |
|  | Access to primary care and optional procedures (such as, colonoscopy and cataract surgery) is limited and health care is only available in urgent and emergency situations. | 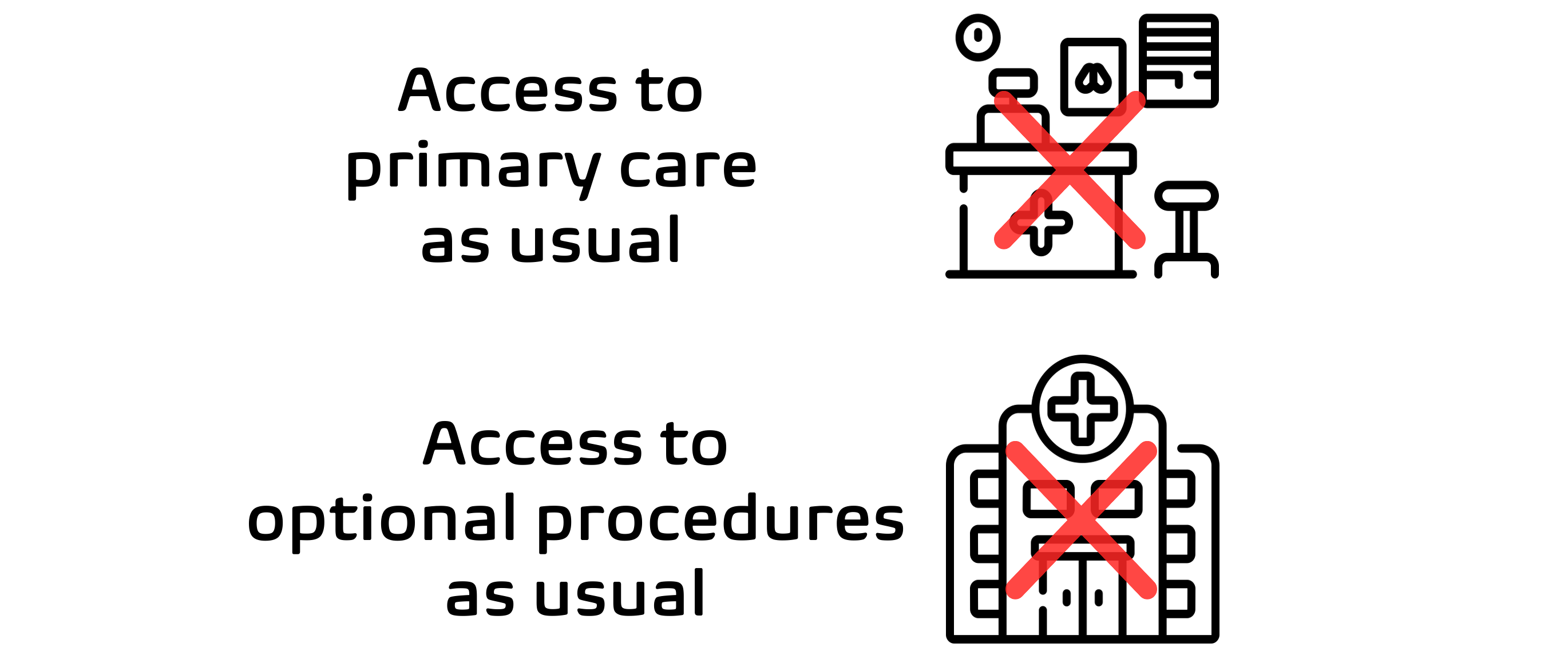 | ^16, 17^ |

1. Supplementary Figures


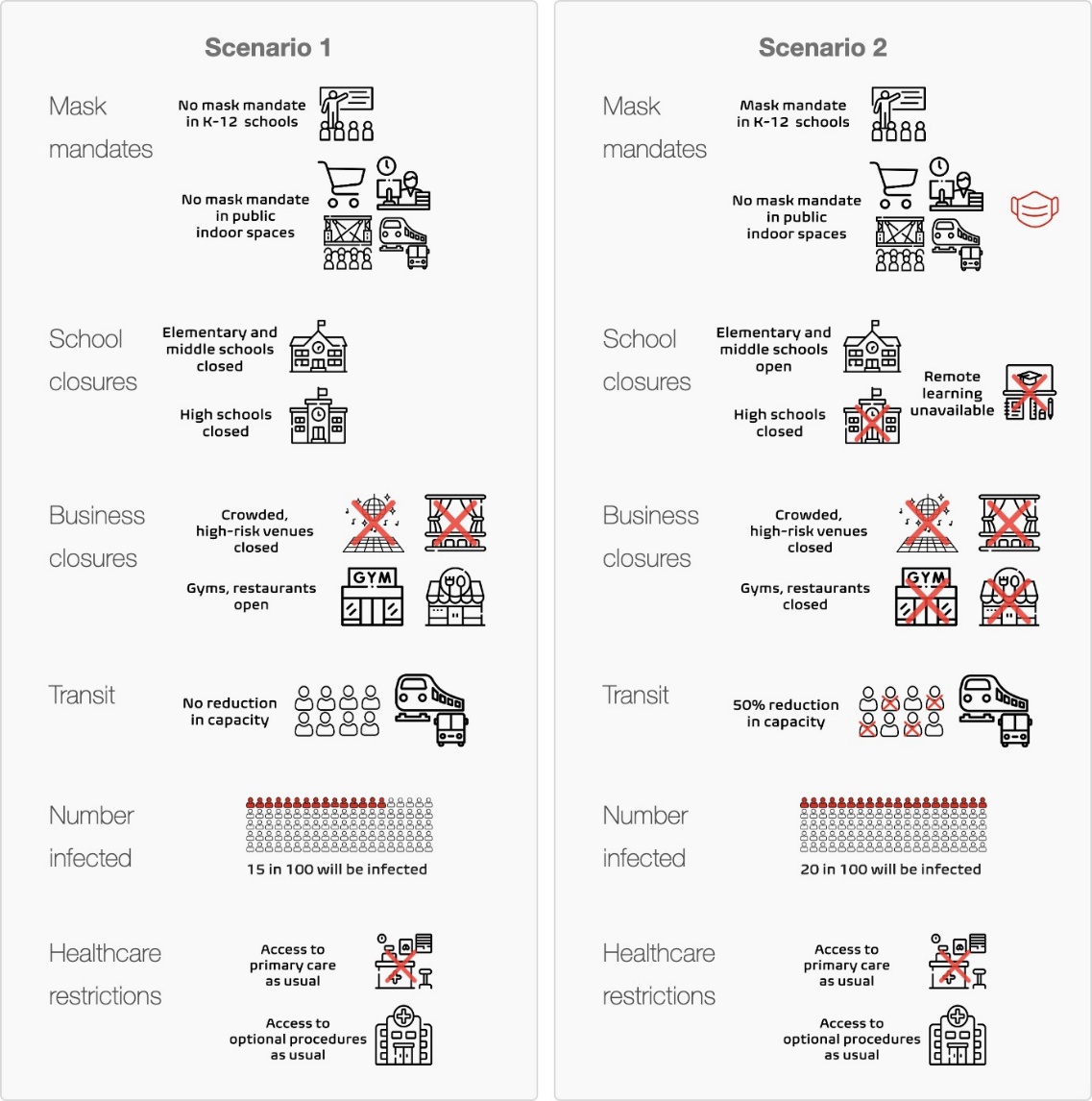


Fig. S1: Sample choice task to select between two scenarios. The participants are provided the following instruction on the top of each choice task: “Put yourself back to a time when COVID-19 was spreading in your county and a vaccine was available. Your county’s public health officials are considering different options for reducing the spread of COVID-19 over the next month. On each of the next pages, we’ll show you two of the options that could be considered to reduce the spread of COVID-19. Of the options shown, select the one that you would rather live under.”


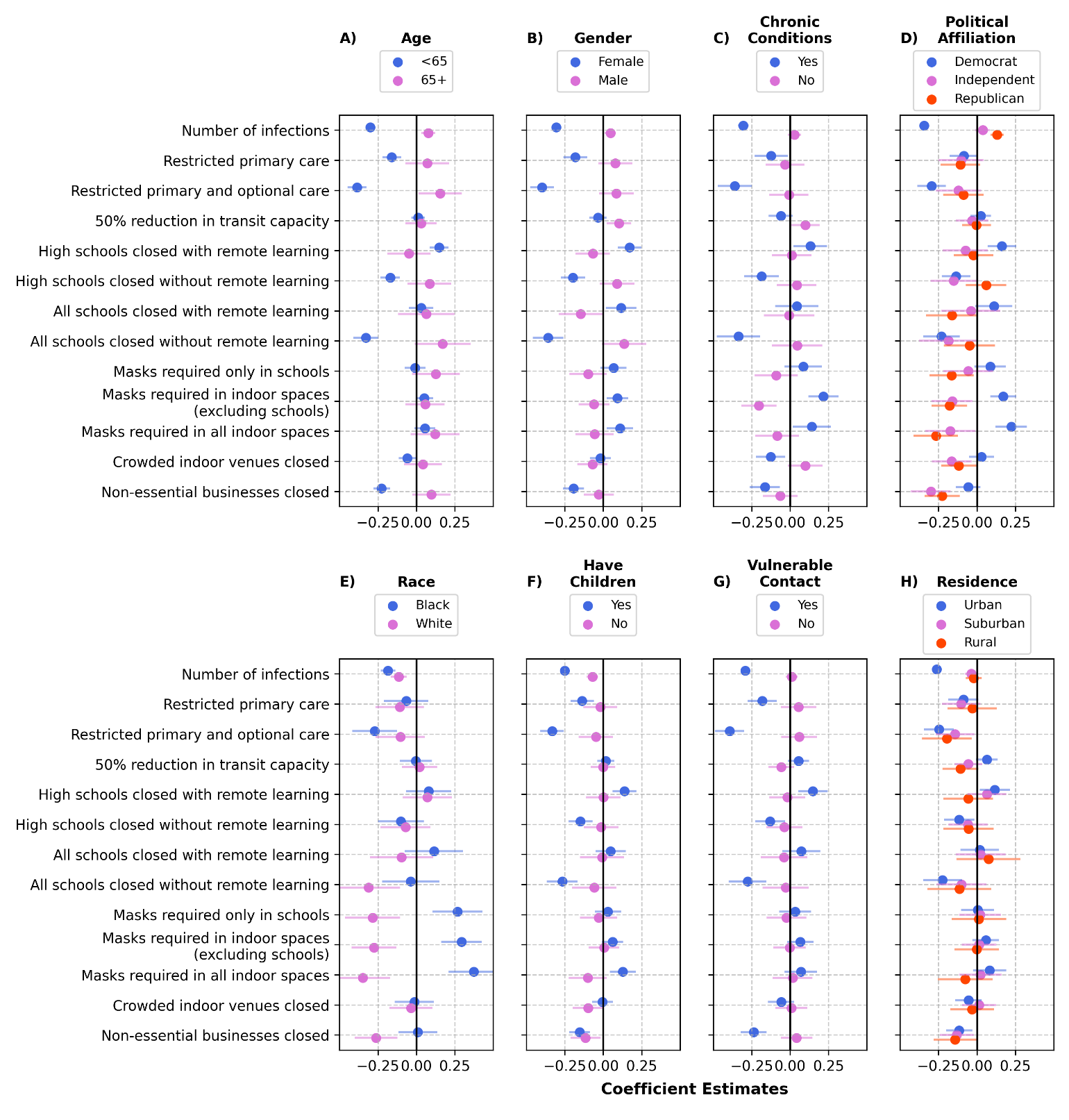


Fig. S2: Coefficient estimates for different subgroups under the survey scenario where an effective vaccine was assumed to be available.


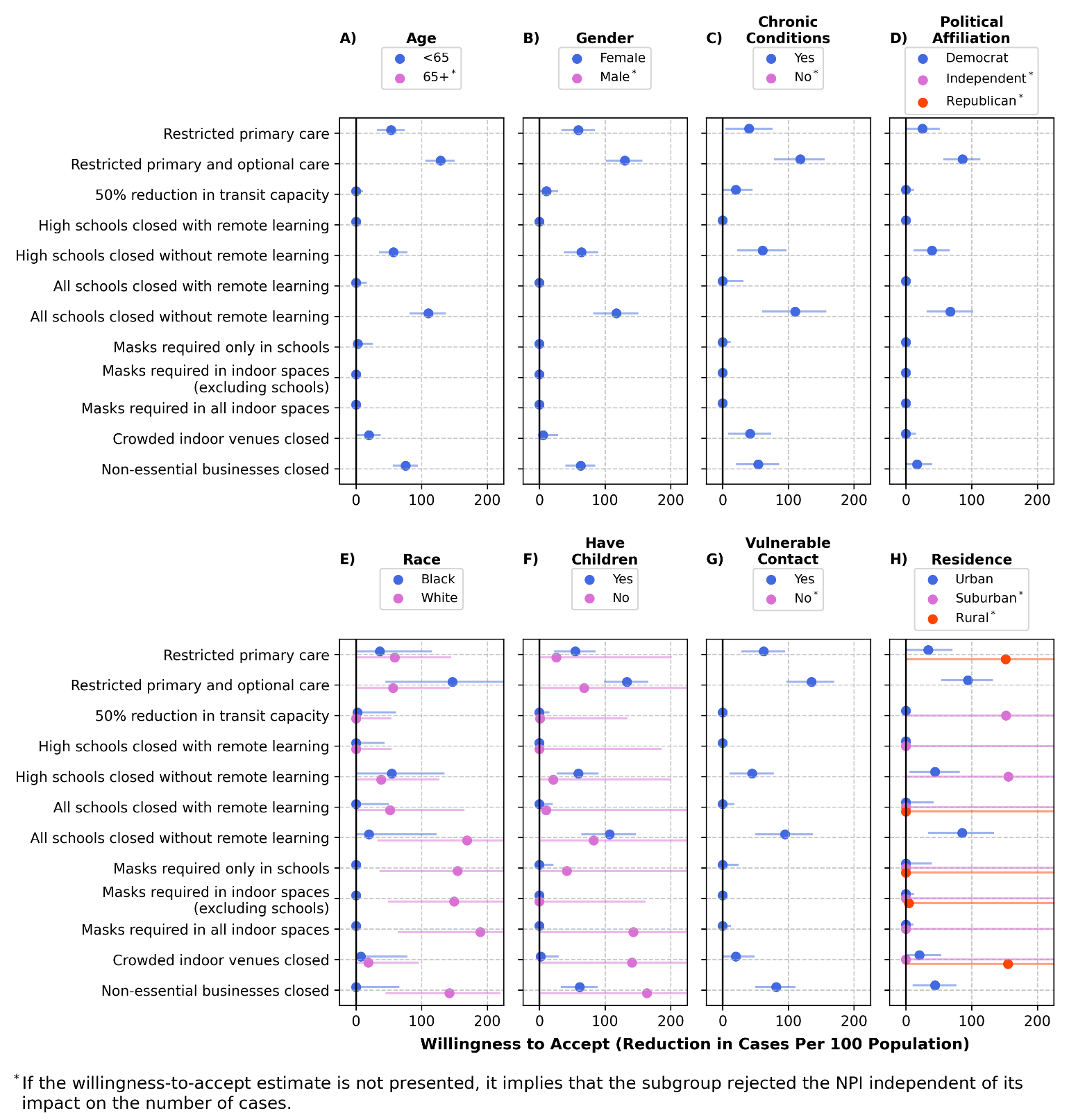


Fig. S3: WTA thresholds for different NPIs among different population subgroups under the survey scenario where an effective vaccine was assumed to be available. WTA=0 implies that the population found the NPI acceptable, even with minimal impact on cases in the next month. If WTA is not presented for a subgroup (e.g., for Male in Panel B), it implies that the subgroup rejected the NPI independent of its impact on the number of cases.

References

[1] Qualtrics. Getting Started with Conjoint Projects. 2025 1/3/2025]Available from: <https://www.qualtrics.com/support/conjoint-project/getting-started-conjoints/getting-started-choice-based/conjoint-analysis-white-paper/>

[2] Train K, Weeks M. Discrete choice models in preference space and willingness-to-pay space: Springer 2005.

[3] U.S. Census Bureau. 2024.

[4] Freepik. Work icons. 2024.

[5] geotatah. Classroom icons. 2024.

[6] Kiranshastry. Shopping cart icons. 2024.

[7] K. P. Arts. Remote learning icons. 2024.

[8] Freepik. High school icons. 2024.

[9] Freepik. Elementary school icons. 2024.

[10] AmethystDesign. Nightclub icons. 2024.

[11] Freepik. Theatre icons. 2024.

[12] Freepik. Gym icons. 2024.

[13] Freepik. Restaurant icons. 2024.

[14] Freepik. Person outline icons. 2024.

[15] Uniconlabs. Public transit icons. 2024.

[16] Freepik. Doctor's office icons. 2024.

[17] Freepik. Hospital icons. 2024.
